# Cyclodextrin Encapsulated pH Sensitive Dyes as Fluorescent Cellular Probes: Self-Aggregation and In Vitro Assessments

**DOI:** 10.3390/molecules25194397

**Published:** 2020-09-24

**Authors:** Monica-Cornelia Sardaru, Oana Carp, Elena-Laura Ursu, Anda-Mihaela Craciun, Corneliu Cojocaru, Mihaela Silion, Vladyslava Kovalska, Ionel Mangalagiu, Ramona Danac, Alexandru Rotaru

**Affiliations:** 1 “Petru Poni” Institute of Macromolecular Chemistry, Romanian Academy, Grigore Ghica Voda Alley 41 A, 700487 Iasi, Romania; monicasardaru13@gmail.com (M.-C.S.); rachita.oana@icmpp.ro (O.C.); ursu.laura@icmpp.ro (E.-L.U.); olaru.anda@icmpp.ro (A.-M.C.); cojocaru.corneliu@icmpp.ro (C.C.); silion.mihaela@icmpp.ro (M.S.); 2 Alexandru Ioan Cuza University of Iasi, Chemistry Department, 11 Carol I, 700506 Iasi, Romania; ionelm@uaic.ro (I.M.); rdanac@uaic.ro (R.D.); 3 Institute of Molecular Biology and Genetics, NASU, 150 Zabolotnogo St., 03143 Kyiv, Ukraine; v.kovalska@gmail.com; 4 Scientific Services Company Otava Ltd., 150 Zabolotnogo St., 03143 Kyiv, Ukraine

**Keywords:** fluorescent indolizines, cyclodextrin inclusion complex, molecular docking, in vitro experiments, organelle markers

## Abstract

We have designed and synthesized a series of novel, supramolecular, long-lived fluorescent probes based on the host-guest inclusion complexes formation between fluorescent indolizinyl-pyridinium salts and β-cyclodextrin. Fluorescence and electrospray ionisation mass spectrometry experiments, supported by theoretical molecular docking studies, were utilized in the monitoring of the inclusion complexes formation, evidencing the appearance of corresponding 1:1 and 1:2 species. Additionally, the influence of the guest molecule over the aggregation processes of the cyclodextrin inclusion complexes was investigated by transmission electron microscopy. The absence of cytotoxicity, cellular permeability, long-lived intracellular fluorescence, and in time specific accumulation within acidic organelles identified the investigated supramolecular entities as remarkable candidates for intracellular fluorescence probes. Co-staining experiments using specific organelle markers revealed the fact that, after a 24-h incubation period, the inclusion complexes accumulate predominantly in lysosomes rather than in mitochondria. This study opens new possibilities for a broad range of fluorescent dyes with solubility and high toxicity issues, able to form inclusion complexes with β-cyclodextrin, to be tested as intracellular fluorescence probes.

## 1. Introduction

The distinctive ability of cyclodextrins (CDs) to form host–guest inclusion complexes with small lipophilic molecules or polymers has attracted the attention of researchers during the last four decades [1,2,3,4,5]. The formation of an inclusion complex where CD plays the role of a host molecule and the suitable hydrophobic entity as guest molecule changes the physicochemical properties of both the guest and the host molecules, typically leading to improved physicochemical and biological properties of the guest entity [6]. CD inclusion complexes, CD pseudo rotaxanes, or rotaxanes have already found their applications in the fields including food and pharmaceutical sciences [7,8,9,10], fluorophores properties improvement [11,12], and catalysis [13,14]. Besides the inclusion complex formation, both CDs and CD inclusion complexes may self-assemble in aqueous solutions into nano or micro aggregates by means of weak hydrogen bonds and hydrophobic forces [11,15,16,17]. In the case of inclusion complexes, the size of the aggregates may range from 100 to 4000 nm, while the shape of the aggregates strongly depends on the guest nature. A large variety of aggregation shapes, including micelles [16], tubes [18], rods [19,20], or discs [21], have been extensively investigated by microscopic methods, such as scanning tunneling microscopy, atomic force microscopy, transmission electron microscopy (TEM), and scanning electron microscopy [22]. The interest in CD complexes aggregates and in the self-aggregation mechanism is explained by their potential applications as candidates for complex drug delivery systems, functional materials, or molecular devices [23,24,25]. It was shown that the formation of CD complexes aggregates with drugs might play an important role in CD enhancement of drug bioavailability [6].

Despite the fact that the reported applications of the CD aggregates refer mostly to the drug delivery area, knowing the key factors influencing the mechanisms for the aggregation formation, stability, and most importantly, the impact of the self-aggregation on the CD complexes properties could open possibilities for their applications in other fields.

In our recent research [26], we have observed a guest specific aggregation of β-CD/indolizinyl-pyridinium salt derivative inclusion complex into long nano and micro crystals. The investigated system represented an aqueous mixture of 1:1 and 1:2 inclusion complexes with improved cytotoxicity properties utilized in long-term fluorescent staining of cell acidic organelles. 

In the current work, we undertake further studies on the influence of the nature of indolizinyl-pyridinium salt derivatives upon the β-CD inclusion complexes self-aggregation as well as the ability of the newly prepared complexes to serve as long-term monitoring non-toxic fluorescent probes [27]. This aspect is particularly important due to the continuous high demand in photostable fluorophores, especially for in vivo microscopy, to obtain high quality images and to allow prolonged monitoring of same cells over time [28]. 

The formation of the β-CD complexes with three new fluorescent indolizinyl-pyridinium salt derivatives was investigated by TEM, electrospray ionization mass spectroscopy (ESI-MS), and fluorescence measurements, supported by molecular docking theoretical studies. Additionally, aqueous solutions of the prepared inclusion complexes were tested as non-toxic fluorescent lysosome and, due to structural similarities with already reported fluorescent probes [29,30], mitochondria staining agents.

## 2. Results and Discussion

### 2.1. Synthesis and Characterization

#### 2.1.1. Synthesis of Indolizinyl-Pyridinium Salts Derivatives

**Scheme 1:**
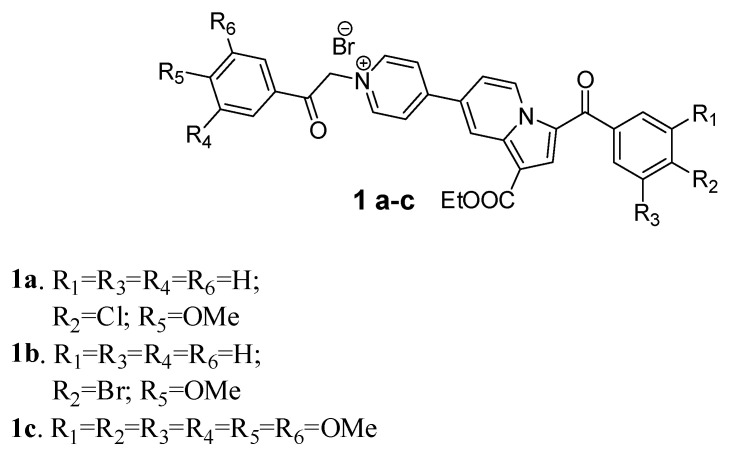
General structure of indolizinyl-pyridinium salts **1**(**a**–**c**).

In order to investigate the influence of the guest indolizinyl-pyridinium salt derivatives on the CD complexes aggregation, and to extend the collection of fluorescent pH sensitive CD host–guest probes, we have synthesized three fluorescent indolizinyl-pyridinium salts **1**(**a**–**c**) (Scheme 1) containing various substituents at the marginal phenyl moieties. Compounds **1**(**a**–**c**) were synthesized in moderate yields in an adopted two step synthesis based on our methodological background in the preparation of substituted indolizines [31,32,33].

We have designed the substituents of the marginal phenacyl moieties in order to be able to observe the influence of the minor (compound **1a** and **b**, possessing correspondingly Cl and Br) and major (compound **1c**, containing six methoxy groups) changes in the guest molecule structure upon the inclusion complex formation and, subsequently, the formation of the corresponding aggregates.

#### 2.1.2. Preparation and Characterization of **1**(**a**–**c**)**_CD** Inclusion Complexes

Indolizinyl-pyridinium salts **1**(**a**–**c**) insoluble in water were tested for the formation of inclusion complexes with β-CD under the guidance of our earlier report on similar system [26]. An equivalent of each compound was suspended in water, followed by the addition of an excess of β-CD (5 eq.) and the suspension was heated to 90 °C until the reaction solution became transparent. Interestingly, the solubilization of **1**(**a**–**c**) compounds was accomplished after 25 min of heating, the solutions remaining completely transparent after cooling down of the reaction mixture to room temperature in case of **1c_CD**, while in case of **1a_CD** and **1b_CD** the formation of slightly cloudy solutions was observed. The increase of the CD amount in the reaction mixture to 10 equivalents in order to push the equilibrium to the formation of stable water-soluble inclusion complexes still did not change the appearance for the **1a_CD** and **1b_CD** at room temperature. The utilization of these solutions in the subsequent analyses was possible after a microfiltration procedure. 

**Figure 1:**
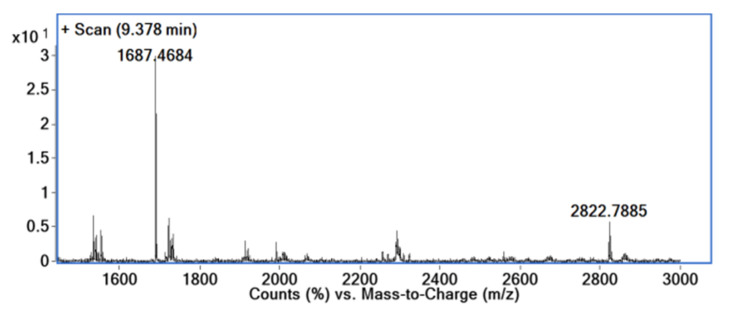
Examples of ESI-MS spectra of the **1a_CD** reaction mixture at 1:5 molar ratio. Peaks corresponding to the formation of 1:1 inclusion complex (M^+^-Br + CD ion at *m*/*z* 1687) and 1:2 (M^+^-Br + 2 CD ion at *m*/*z* 2822) have been identified.

To determine the formation of the inclusion complexes, ESI-MS experiments were performed for all **1**(**a**–**c**)**_CD** water solutions, this method being frequently utilized in studying the inclusion phenomena [34,35] and subsequent determination of the stoichiometry of the complexes [36] (Figure 1, Supplementary Figures S1–S3). Representative ESI-MS spectrum for compound **1a_CD** (Figure 1) as well as spectra for compounds **1b_CD** and **1c_CD** (Figures S2 and S3) have shown characteristic peaks corresponding to the formation of CD inclusion complexes.

Thus, the investigated inclusion complexes samples revealed the presence of CD molecule MNa^+^ ion at *m*/*z* 1157.3 (Supplementary Figures S2 and S3) and, most importantly, in all cases the signals corresponding to 1:1 (M^+^-Br + CD) and 1:2 (M^+^-Br + 2 CD) inclusion complexes were observed and assigned (Supplementary Figures S1–S3).

**Figure 2:**
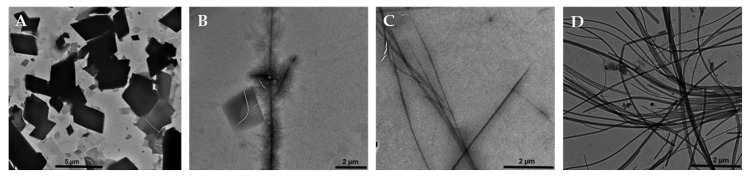
Examples of TEM images for: (**A**) trapezoidal-like sheets of β-CD; (**B**) branched needle-like aggregates **of 1a_CD**; (**C**) needle-like aggregates of **1b_CD**; (**D**) wire-like assemblies of **1c_CD**.

Transmission electronic microscopy images were recorded to additionally confirm the formation of inclusion complexes of the investigated compounds (Figure 2, Supplementary Figure S4), and to monitor the influence of the guest molecule nature to impact the aggregation of the complexes.

In order to confirm the impact of the investigated guest molecules upon the CD aggregation, we first measured the TEM images of CD (Figure 2A). The images of β-CD revealed the formation of the characteristic trapezoidal-like sheets of different micrometric size depending on the utilized concentration of the CD solution. The analyzed TEM images of the inclusion complexes (Figure 2B–D) indicated dramatic changes in the shape of the corresponding aggregates and clear influence of the structure of the guest molecule over the process. Thus, the images of compound **1a_CD** (Figure 2B, Supplementary Figure S4A) revealed the formation of the micrometer size wires with the length not exceeding several micrometers, possessing multiple brunches and ramifications along the core wires. In the case of **1b_CD**, similar wire-like structures were observed in terms of the size of the wires, but the absence of the branches and ramifications was clearly noticed (Figure 2C, Supplementary Figure S4B), revealing a somewhat different aggregation mechanism induced by the bromine moiety (**1b**) over the chlorine one (**1a**). Additionally, in the case of **1b_CD,** the presence of multiple wires of much smaller diameter was also observed in the background (Supplementary Figure S4B). A complete change in the formation of the aggregates was observed in the case of **1c_CD** (Figure 2D, Supplementary Figure S4C). Uniform high-contrast wires with the length exceeding tens of micrometers could be observed all over the investigated surface without observed branches or thin structures in the background. In this case, the substituents of compound **1c** strongly impact the crystallization process of the inclusion complex, facilitating the assembly of small aggregates into long wire-like structures by means of intermolecular hydrogen bonding with nearby inclusion complexes. The presence of three methoxy groups of the guest molecule may strongly affect the head–head, tail–tail, or head–tail cyclodextrin arrangements by the formation of multiple OH–O bonds where methoxy oxygen serves as acceptor [37].

Interestingly, the inclusion complexes aggregations in aqueous solutions typically result in large micelle-like structures [22], micrometer-sized rods [20,38,39], or spherical shape nanoaggregates [15], while the formation of wires is more distinctive to unmodified cyclodextrins [21].

Absorption and fluorescence spectra of **1**(**a**–**c**) and **1**(**a**–**c**)**_CD** were recorded at different pH values (1.0, 7.4, and 13.0) and the results compared. The absorption spectra of **1**(**a**–**c**) at pH = 1.0 (Supplementary Figure S5) consist of two bands appearing around 290 nm (slightly structured in case of **1a** and **b**) and 410 nm. With the increase of the pH value to 7.4, additional band appears around 510 nm only in the case of **1a** and **b**, while the spectrum of compound **1c** didn’t suffer major changes. At the pH value of 13.0, all the investigated compounds exhibited similar spectra consisting of two broad bands around 290 and 500 nm. In the case of **1**(**a**–**c**)**_CD** (Supplementary Figure S5), all the investigated compounds have shown two intense bands at 290 and 410 nm in similar spectra at pH values of 1.0 and 7.4, indicating different behavior when compared to starting **1**(**a**–**c**). Major changes in the shape of the spectra at pH = 13.0 were observed for all **1**(**a**–**c**)**_CD** both in comparison to the **1**(**a**–**c**) and to each other. Compound **1a_CD** presented a major band at 490 nm similar in intensity to the band at 390 nm. Compound **1b_CD** also showed the band at 390 nm and an intense band at 410 nm with a shoulder at 490 nm. Compound **1c_CD** on the other hand consisted of an intense band at 390 nm and a sharp band at 490 nm with much higher intensity than the one at 390 nm. 

**Figure 3:**
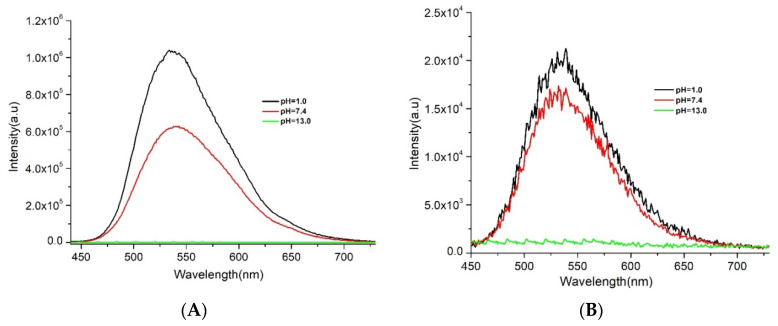
Fluorescence spectra of compounds **1a** (**A**) and **1a_CD** (**B**) at pH = 1.0 (0.1 M HCl, black); 7.4 (1× TAE buffer, red) and 13.0 (0.1M NaOH, green).

The fluorescence spectra of **1**(**a**–**c**) and **1**(**a**–**c**)**_CD** were measured by excitation at 420 nm at different pH values (Figure 3, Supplementary Figure S6) due to the pH switchable fluorescence nature of compound **1**. 

Compounds **1**(**a**–**c**) exhibited similar behaviour furnishing relatively strong signals around 550 nm at acidic pH, with an approximately 50% or higher drop in intensity at neutral pH value, followed by low to no intensity at pH = 13.0 (Figure 3A, Supplementary Figure S7A,C). The pH-dependent behaviour may be explained by the reversible transformation of pyridinium salts into analogous non-fluorescent ylides [40]. The inclusion complexes **1**(**a**–**c**)**_CD,** on the other hand, demonstrated slightly different pH dependent behaviours (Figure 3B, Supplementary Figure S7B,D), showing a similar low intensity at basic pH values, but changed intensities at pH = 1.0. In the case of compounds **1a** and **b**, the fluorescence intensity at acidic pH showed values only slightly higher than intensities at pH = 7.4, while in the case of **1c**, the fluorescence intensities at both the acidic and neutral pH were comparable. The decrease of fluorescence intensity of the inclusion complexes at pH = 1.0 could be explained by the formation of the inclusion complex, thus inducing specific rotation limitations of the indolizinyl-pyridinium salt molecules due to the steric hindrances induced by the CD.

### 2.2. Molecular Docking Studies

**Figure 4:**
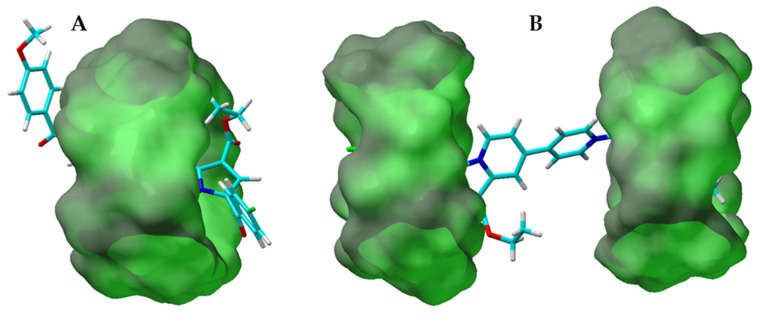
Examples of molecular docking models of compound **1a** in complex with β-CD showing the possibility of the 1:1 (**A**) and 1:2 (**B**) inclusion complexes formation**.**

In order to support experimental results obtained during fluorescence and TEM investigations and to approve the possibility for the formation of both 1:1 and 1:2 inclusion species observed in ESI-MS experiments, molecular modeling studies by means of docking protocol were conducted. The calculations were performed by the AutoDock Vina method implemented in the YASARA Structure software package [41]. The analysed β-CD and compounds **1**(**a**–**c**) were first constructed and optimized at PM3 level of theory using Hyperchem software [42] and subsequently exported to YASARA program. After optimization, the molecular docking (MD) simulations indicated the formation of both 1:1 and 1:2 complexes of compounds **1**(**a**–**c**) with β-CD (Figure 4, Supplementary Figures S8 and S9).

In the case of 1:1 complexes, all the investigated compounds were included inside the cyclodextrin cavity adopting similar semi-folded conformation, with the cyclodextrin molecule covering the most part of the compounds’ inner 4,4′-bipiridyl moiety (Figure 4A, Supplementary Figures S8A and S9A). The theoretically calculated binding energy (E_b_) for the optimized 1:1 inclusion complexes (Table 1) were comparable, ranging between −6.11 to −6.70 kcal/mol, while the dissociation constant (K_d_) showed similar data for compounds **1a** and **1b** (17.225 and 11.983 µM correspondingly). For the optimized **1c** 1:1 inclusion complexes, the K_d_ value was noticeably higher (33.494 µM), revealing the greater stability of 1:1 complexes for compounds **1a** and **1b** over compound **1c**.

Analysing the images for the optimized 1:2 complexes (Figure 4B, Supplementary Figures S8B and S9B), similarities in the structures of the **1a_CD** and **1b_CD** complexes were clearly observed. Two CD molecules were positioned on the peripheral aromatic moieties of the guest molecules, completely covering these parts of the molecule. In the case of **1c_CD**, the two CD molecules were positioned slightly different (Supplementary Figure S9B), one CD was completely embedding the marginal indolizinic trimethoxy-phenyl moiety, while the other CD was implanted deeper towards the centre of the guest, partly covering the trimethoxy-phenyl and most of the quaternary pyridine moiety, leaving the protruding three methoxy groups on the outer side of CD. This optimized 1:2 complex of compound **1c_CD** supports the TEM results for the formation of long wires. The protruding three methoxy groups might benefit the multiple complexes intermolecular binding by the formation of additional hydrogen bonds between the methoxy groups and the H/OH cyclodextrin groups of the other complex side, resulting in uniform and linear assembling.

The E_b_ values for the optimized 1:2 inclusion complexes (Table 1) were similar for all the investigated compounds (between −7.84 and −7.34 kcal/mol) and close to the values for the 1:1 complexes. In the case of K_d_, values for compounds **1a**, **1b**, and **1c** were considerably lower (by factor 6, 7, and 8 correspondingly) when compared to the values for 1:1 complexes, indicating the greater stability of 1:2 complexes.

### 2.3. In Vitro Cell Experiments

#### 2.3.1. Cytotoxicity Assessments

**Figure 5:**
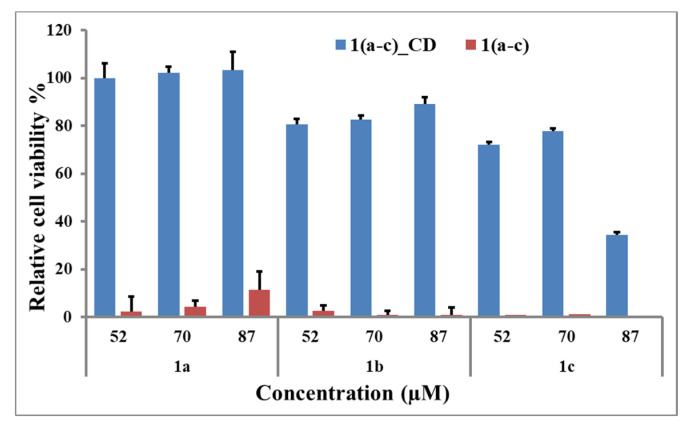
In vitro cell viability (MTS assay) results for compounds **1**(**a**–**c**) (red) and **1**(**a**–**c**)**_CD** (blue) at 52 µM, 70 µM, 87 µM of corresponding indolizinyl-pyridinium salts on HeLa cell line.

The cell staining experiments with compounds **1**(**a**–**c**)**_CD** started with their cytotoxicity evaluation and comparison with the initial **1**(**a**–**c**)**,** using a 3-(4,5-dimethylthiazol-2-yl)-5(3-carboxymethonphenol)-2-(4-sulfophenyl)-2H-tetrazolium (MTS) (Figure 5) on cervical cancer HeLa cells (HeLa). Solutions of compounds **1**(**a**–**c**) at concentrations equal to 52 μM, 70 μM, and 87 μM and β-cyclodextrin inclusion complexes **1**(**a**–**c**)**_CD** solutions containing corresponding indolizinyl-pyridinium salts at similar concentrations have been incubated with HeLa cells and evaluated. The obtained results revealed the fact that the cell viability was significantly enhanced by the formation of the inclusion complexes especially in case of **1a_CD** and **1b_CD** (cell viability above 75%) for all the investigated concentrations. Slightly lower values were obtained for **1c_CD,** especially at higher tested concentrations. The cytotoxicity of free β-CD was not evaluated in this experiment due to the earlier reported data on the absence of β-CD toxicity at concentrations up to 3.3 mM on HeLa cell line [43].

The starting compounds **1**(**a**–**c**) displayed strong toxicity at all the investigated concentrations, the cell viability values not exceeding 10%. The obtained results are in accordance with the previously reported high toxicity of pyridinium salts [44,45] and our recent observations on toxicity attenuation after the formation of the inclusion complexes [26,46].

#### 2.3.2. Staining of Living Cells Using **1**(**a**–**c**)_**CD** solutions

**Figure 6:**
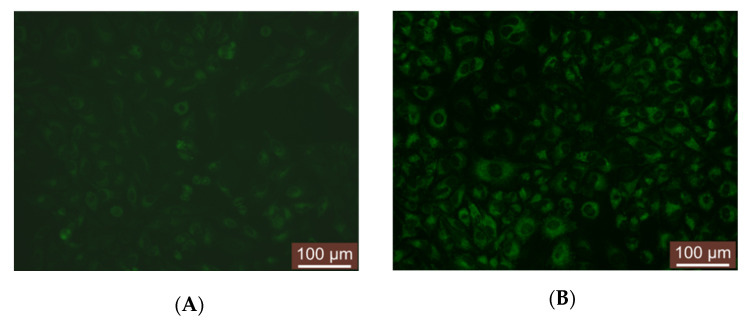
Examples of images for compound **1a_CD** uptake into HeLa cells after 15 min (**A**) and 24 h (**B**) incubation.

Solutions of compounds **1**(**a**–**c**)**_CD** were assessed for their ability to penetrate the cell membrane and for localization and staining of cell components in living cells by fluorescence microscopy. HeLa cells were incubated with 52-µM water solutions of **1**(**a**–**c**)**_CD** and imaged simultaneously over 15 min and 24 h with an inverted Leica DMI 3000 B microscope with fluorescence GFP filter (Figure 6).

**Figure 7:**
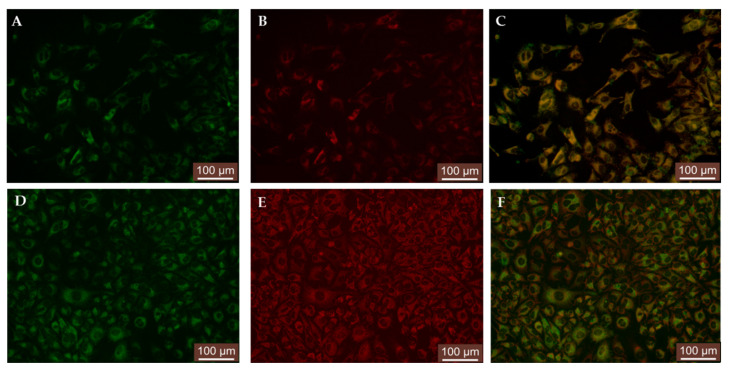
Examples of images for intracellular distribution of compound **1a_CD** after 24 h (**A**,**D**) compared to LysoTracker Red (**B**) and MitoTracker Red (**E**), including corresponding overlays (**C**,**F**).

Following 15 min incubation with the investigated fluorescence solutions, clearly visible intracellular and extranuclear localization was observed for all samples (Figure 6A, Supplementary Figures S10A and S11A). Subsequent incubation of the probe-loaded cells for 24 h revealed a strong increase in intracellular fluorescent signal (Figure 6B, Supplementary Figures S10B and S11B) accompanied by particular localized agglomerations within the cytoplasm. These specific cellular localizations of **1**(**a**–**c**)**_CD** were further examined using specific organelle markers. The observed increase in agglomerations fluorescent signal and the distribution pattern within the cytoplasm suggested compounds’ localization in either cell acidic vesicles or mitochondria. Consequently, to check the specificity of **1**(**a**–**c**)**_CD** to selectively stain corresponding organelles, parallel co-staining experiments in HeLa cells with LysoTracker and MitoTracker were undertaken (Figure 7, Supplementary Figures S12–S15).

HeLa cells incubated for 24 h with **1**(**a**–**c**)**_CD** (Figure 7A,D, Supplementary Figures S12A and S13A) were next treated with LysoTracker Red for 30 min and imaged to give a distinct lysosomal labelling pattern (Figure 7B, Supplementary Figures S12B and S13B). The overlay analysis of the images outcomes suggested the localization of inclusion complexes in lysosomes (Figure 7C, Supplementary Figures S12C and S13C), the results being similar to the previously reported inclusion complex [26].

The similarly performed co-staining experiment with MitoTracker Red on 24 h incubated HeLa cells with **1**(**a**–**c**)**_CD** resulted in similar outcomes (Figure 7D–F, Supplementary Figures S14A–C and S15A–C), the inclusion complexes exhibited slightly lower mitochondrial distribution and localization (Figure 7F, Supplementary Figures S14C and S15C). To support the observations obtained from the visual assessment, we applied the colocalization analysis of the dyes using Fiji software which was designed for image processing and having implemented different plugins that can give information about Pearson’s correlation coefficient (PCC) [47]. The PCC values between compounds **1**(**a**–**c**)**_CD** and LysoTracker or MitoTracker were obtained by applying two different plugins of the Fiji software (JACoP and Coloc 2) and are presented in Table 2.

Pearson’s correlation analysis of whole images resulted in very high Pearson coefficient values in the range of 0.5–0.8 for the LysoTracker co-staining experiments, and high values (0.4–0.6) for the MitoTracker dye. Only insignificant differences between the outcomes obtained by the two utilized plugins (JACoP and ColoC) in case of all utilized compounds and the involved commercial stains were obtained. The obtained and compared PCC values indicated major differences only in case of compound **1a_CD** between the two colocalization experiments, while for the **1b_CD** and **1c_CD** the data were comparable. Generally, the PCC outcomes suggest the fact that compounds **1**(**a**–**c**)**_CD** are able to accumulate and yield strong fluorescent signal predominantly in lysosomes due to their acidic nature. On the other hand, these data also suggest in time accumulation of the investigated compounds in mitochondria but less efficiently due to, probably, the non-acidic nature of mitochondria.

## 3. Materials and Methods

Commercially available chemical reagents were supplied by Sigma-Aldrich (Schnelldorf, Germany) and used without further purification. HeLa (human cervix adenocarcinoma) cells were acquired from CLS-Cell-Lines-Services-GmbH (Heidelberg, Germany). An MTS assay (CellTiter 96 AQueous One Solution Cell Proliferation Assay) was acquired from Promega (Madison, WI, USA), Mito Tracker Red CMXRos was acquired from Thermo Fisher (Eugene, OR, USA) and LysoTracker Red DND-99 was acquired from Invitrogen (Eugene, OR, USA). Ultrapure water obtained from a commercial Millipore system was used for preparing the working solutions. Dimethyl sulfoxide (DMSO) used for the spectrometric studies was of spectroscopic grade.

### 3.1. Procedure for the Synthesis of Compounds ***1***

Compounds **1** were synthesized and characterized according to previously reported procedures [44,48]. 4-(3-(4-chlorobenzoyl)-1-(ethoxycarbonyl)indolizin-7-yl)-1-(2-(4-methoxyphenyl)-2-oxoethyl)pyridin-1-ium bromide **1a** (MW: 633.92 g/mol). Yield: 93%. All spectral data are in agreement with the literature report [44]. 4-(3-(4-bromobenzoyl)-1-(ethoxycarbonyl)indolizin-7-yl)-1-(2-(4-methoxyphenyl)-2-oxoethyl)pyridin-1-ium bromide **1b** (MW: 678.37 g/mol). Yield: 92%. All spectral data are in agreement with the literature report [44]. 4-(1-(ethoxycarbonyl)-3-(3,4,5-trimethoxybenzoyl)indolizin-7-yl)-1-(2-oxo-2-(3,4,5-trimethoxyphenyl)ethyl)pyridin-1-ium bromide **1c** (MW: 749.60 g/mol). Yield: 70%. All spectral data are in agreement with the literature report [48].

### 3.2. Procedure for the Preparation of Compounds ***1***(***a***–***c***)***_CD***

Compounds **1**(**a**–**c**) (0.01 mmol) and β-CD (0.05 mmol, 56.7 mg) were suspended in distilled water (2.5 mL) in a vial and stirred at 90 °C for 40 min until the reaction mixture became transparent. After cooled to room temperature, the transparent solution of **1c_CD** was used for analyses and subsequent experiments, while in the case of **1a_CD** and **1b_CD,** slightly cloudy solutions were filtered out using Phenex syringe filters with pore size 0.45 µm.

### 3.3. Characterisation Techniques

UV/Vis spectra were recorded on a Lambda 35 UV/Vis spectrophotometer (PerkinElmer, Waltham, MA, USA) using cuvettes with a sample volume of 3000 μL (wavelength range 200−1000 nm). Analyzed samples **1**(**a**–**c**) were first dissolved in dimethylsulphoxide (DMSO) to make 4-mM stock solutions. Samples were measured at different pH values by diluting 10 μL of stock solution in 1990 μL NaOH (0.1 M) for Ph = 13.0, 10 μL of stock solution in 1990 μL HCl (0.1 M) for pH = 1.0, and 10 μL of stock solution in 1990 μL 1× TAE buffer solution (tris, 40 mM; EDTA, 2 mM; adjusted to pH 7.4 with acetic acid) for pH = 7.4. Samples **1**(**a**–**c**)**_CD** were measured by diluting 10 μL of corresponding reaction mixture, containing 4 mM equivalent concentration of **1**(**a**–**c**) in 1990 μL NaOH (0.1 M) for pH = 13.0, 10 μL in 1990 μL HCl (0.1 M) for pH = 1.0, and 10 μL in 1990 μL 1× TAE buffer solution (tris, 40 mM; EDTA, 2 mM; adjusted to pH 7.4 with acetic acid) for pH = 7.4.

Fluorescence spectra were registered on a Fluoromax 4 fluorescence spectrophotometer (Horiba Scientific, Longjumeau, France) using cuvettes with a sample volume of 3000 μL (excitation at 415 nm and emission range between 430 nm and 700 nm). Samples **1**(**a**–**c**) were first dissolved DMSO to make 4-mM stock solutions. Samples were measured at different pH values by diluting 1 μL of stock solution in 1999 μL NaOH (0.1 M) for pH = 13.0, 1 μL of stock solution in 1999 μL HCl (0.1 M) for pH = 1.0, and 1 μL of stock solution in 1999 μL 1× TAE buffer solution (tris, 40 mM; EDTA, 2 mM; adjusted to pH 7.4 with acetic acid) for pH = 7.4. Samples **1**(**a**–**c**)**_CD** were measured by diluting 1 μL of reaction mixture, containing 4 mM equivalent concentration of **1**(**a**–**c**) in 1999 μL NaOH (0.1 M) for pH = 13.0, 1 μL of corresponding reaction mixtures in 1999 μL HCl (0.1 M) for pH = 1.0, and 1 μL of reaction mixture in 1999 μL 1× TAE buffer solution (tris, 40 mM; EDTA, 2 mM; adjusted to pH 7.4 with acetic acid) for pH = 7.4. Mass spectrometry data were acquired using an Agilent 6520 Series Accurate-Mass Quadrupole Time-of-Flight (Q-TOF) LC/MS (Agilent, Santa Clara, CA, USA) connected directly to ionization source via mass spectrometer electrospray. The ESI-MS experimental conditions included: ESI in a positive mode, drying gas debit (N2) 9L/min, gas temperature 325 °C; nebulizer pressure 25 psi, capillary voltage 4200 V; fragmentation voltage 200 V; compounds were investigated in a field of *m*/*z* 1000–3000. Transmission electron microscopy (TEM) analysis of the samples was performed on Hitachi HT7700 (Hitachi, Tokyo, Japan) microscope operating at 100 kV in High Resolution Mode. β-CD (87 μM) and **1**(**a**–**c**)**_CD** solutions (3 µL) were deposited on 300 mesh carbon-coated copper grids and dried overnight at room temperature before examination.

### 3.4. Cell Cultures

HeLa cells (from CLS-Cell-Lines-Services-GmbH, Heidelberg, Germany) were cultivated in tissue culture flasks with alpha-MEM medium (Lonza, Basel, Switzerland) supplemented with 10% fetal bovine serum (FBS, Biochrom GmbH, Germany) and a 1% penicillin-streptomycin-amphotericin B mixture (10 K/10 K/25 μg in 100 mL, Lonza). The medium was changed with a fresh one, once in every 3 or 4 days. Once confluence was reached, the cells were washed with phosphate buffered saline (PBS, Invitrogen, Paisley, Scotland, UK), detached with a 1× Trypsin-EDTA mixture (Lonza) followed by the addition of complete growth medium, centrifuged at 200× *g* for 3 min and subcultured into new tissue culture flasks.

An in vitro cell viability study (MTS assay) was performed using the CellTiter 96 Aqueous One Solution Cell Proliferation Assay (Promega, Madison, WI, USA) following the manufacturer’s protocol. HeLa cells were seeded at a density of 10^4^ cells per well in 96 well plates in complete medium (alpha-MEM medium (Lonza) supplemented with 10% fetal bovine serum (FBS) and a 1% penicillin-streptomycin-amphotericin B mixture (10 K/10 K/25 μg in 100 mL, Lonza)). After 24 h, the cells were treated with the indolizinyl-pyridinium salts **1**(**a**–**c**) at concentrations equal to 52 μM, 70 μM, and 87 μM in 100 μL of medium and β-cyclodextrin inclusion complexes **1**(**a**–**c**)**_CD** solutions containing corresponding indolizinyl-pyridinium salts at equivalent concentrations equal to 52 μM, 70 μM and 87 μM in 100 μL of medium, with subsequent incubation at room temperature for another 24 h. Next, 20 μL of CellTiter 96^®^ Aqueous One Solution reagent were added to each well, and the plates were incubated for 1 h before reading the result. Absorbance at 490 nm was recorded with a microplate reader (BMG LABTECH) (Ortenberg, Germany). A blank absorbance value from wells without cells but treated with MTS and with or without the inclusion complex was subtracted from the corresponding absorbance values. Cell viability was calculated and expressed as a percentage relative to the viability of untreated cells which served as the negative control for the inclusion complex. Cells incubated with 0.5% DMSO served as the negative control for the indolizinyl-pyridinium **1**(**a**–**c**) salts. Experiments were performed in three replicates and repeated three times. Data are presented as mean ± S.D.

#### 3.4.1. Cell staIning with the Indolizinyl-Pyridinium Salt and β-Cyclodextrin Inclusion Complex and Co-Staining with LysoTracker Red DND-99

The HeLa cells were incubated with different concentrations of the indolizinyl-pyridinium salts–β-cyclodextrin inclusion complexes (at concentrations equal to 52 μM, 70 μM, 87 μM) and imaged 15 min and at 24 h with an inverted microscope Leica DMI 3000 B equipped with a fluorescence GFP filter. Subsequently, the cells stained with the indolizinyl-pyridinium salt–β-cyclodextrin inclusion complex were treated with 65 nM solution of LysoTracker Red DND-99 (Invitrogen) for 30 min at 37 °C in 5% CO_2_. The cells were then washed three times in PBS and imaged in fresh cell culture medium using an inverted microscope Leica DMI 3000 B equipped with GFP and N2.1 filters.

#### 3.4.2. Cell Staining with the Indolizinyl-Pyridinium Salt and β-Cyclodextrin Inclusion Complex and Co-Staining with MitoTracker^®^ Red CMXRos

The HeLa cells were incubated with different concentrations of the indolizinyl-pyridinium salts and β-cyclodextrin inclusion complexes (at concentrations equal to 52 μM, 70 μM, and 87 μM) for 24 h and then imaged at 15 min and at 24 h with an inverted microscope Leica DMI 3000 B equipped with a fluorescence GFP filter. Subsequently, the cells stained with the indolizinyl-pyridinium salt and β-cyclodextrin inclusion complex were treated with 100 nM solution of MitoTracker^®^ Red CMXRos for 30 min at 37 °C in 5% CO_2_. After staining, the cells were washed three times in PBS and imaged in fresh cell culture medium using an inverted microscope Leica DMI 3000 B equipped with GFP and N2.1 filters.

#### 3.4.3. Colocalization Analysis Based on Pearsons’ Coefficient

All the images subjected to colocalization analysis were used as they were acquired from microscope without any enhance processing. The two images from the same compound (green channel and red channel) were subjected to same analysis steps in Fiji software. Briefly, image color channels were divided in red, green, and blue, and we kept only the color channel which was specific for dye color (green for compounds **1**(**a**–**c**)**_CD** and red for commercial dyes). Then, the obtained images containing only the desired color channel (red and green) were analyzed, in terms of correlation to obtain the values for Pearson’s coefficient (r), by using the JACoP and Coloc 2 plugins available in Fiji software. Importantly, the threshold used was left in automatic mode. The results obtained from these analyses were expressed as they were obtained without other processing steps.

## 4. Conclusions

Three fluorescent indolizinyl-pyridinium salts/β-cyclodextrin inclusion complexes have been prepared and characterized. Fluorescence analysis and ESI-MS experiments have provided evidences for the formation of 1:1 and 1:2 host guest species, while TEM analysis have revealed strong influence of the guest molecules over the aggregation process. The molecular docking studies supported the experimental data on the formation of 1:1 and 1:2 host–guest species and provided structural insights into the possible aggregation mechanisms. Thus, among the investigated **1**(**a**–**c**)**_CD**, the inclusion complex with marginal three methoxyphenyl moieties showed somewhat different organisations and, conceivably, the protruding methoxy groups within the 1:2 inclusion complex might be involved in the hydrogen bonding between the inclusion complexes, leading to wire-like organisations observed in TEM images.

The obtained inclusion complexes have shown similar behaviour in terms of cytotoxicity and cell staining experiments on HeLa cells with the previously reported indolizinyl-pyridinium salt/β-cyclodextrin inclusion complex in terms of the absence of cytotoxicity, cellular permeability, and long-lived intracellular fluorescence, together with the specific accumulation within acidic organelles or mitochondria. Co-staining experiments with lysosome and mitochondria specific markers revealed the fact that the investigated inclusion complexes prefer lysosomal accumulations over the mitochondrial and, unlike commercially available lysosomal markers, no decrease in fluorescent signal was observed. Contrarily, a strong increase in fluorescence over the 24 h period was examined, demonstrating outstanding inclusion complex fluorescent signal stability inside living cells. The proposed supramolecular cyclodextrin-based method in the preparation of fluorescent markers may represent a new approach in the fabrication of novel fluorescent probes in a wide spectral range.

## Figures and Tables

**Table 1:** Theoretically calculated binding energy (E_b_) and dissociation constant (K_d_) for the 1:1 and 1:2 cyclodextrin inclusion complexes of compounds **1**(**a**–**c**).

Compound	Complex 1:1	Complex 1:2
1a	E_b_ = −6.50 kcal/mol K_d_ = 17.225 µM	E_b_ = −7.60 kcal/mol K_d_ = 2.704 µM
1b	E_b_ = −6.71 kcal/mol K_d_ = 11.983 µM	E_b_ = −7.84 kcal/mol K_d_ = 1.803 µM
1c	E_b_ = −6.11 kcal/mol K_d_ = 33.494 µM	E_b_ = −7.34 kcal/mol K_d_ = 4.151 µM

**Table 2:** Pearsons correlation coefficient for the colocalization analysis of compounds **1**(**a**–**c**)**_CD** and commercial dyes LysoTracker or MitoTracker, calculated by Fiji software using JACoP and ColoC plgins.

Commercial Dye	Compound	Pearsons Correlation Coefficient (r)	
		JACoP	Coloc 2
LysoTracker	1a_CD	0.876	0.88
	1b_CD	0.654	0.65
	1c_CD	0.567	0.57
MitoTracker	1a_CD	0.472	0.47
	1b_CD	0.626	0.63
	1c_CD	0.532	0.53

## Supplementary Materials

The following are available online, Figure S1: Examples of ESI-MS spectra of the **1a_CD** at 1:5 molar ratio between indolizine **1a** and CD, Figure S2: Examples of ESI-MS spectra of the **1b_CD** at 1:5 molar ratio between indolizine **1b** and CD, Figure S3: Examples of ESI-MS spectra of the **1c_CD** at 1:5 molar ratio between indolizine **1c** and CD, Figure S4: Examples of TEM images for compounds **1a_CD** (**A**); **1b_CD** (**B**); **1c_CD** (**C**), Figure S5: UV-Vis spectra of compounds **1**(**a**–**c**) at pH value of 1.0 (0.1 M HCl), 7.4 (1× TAE) and 13.0 (0.1 M NaOH), Figure S6: UV-Vis spectra of compounds **1**(**a**–**c**)**_CD** at pH value of 1.0 (0.1 M HCl), 7.4 (1× TAE) and 13.0 (0.1 M NaOH), Figure S7: Fluorescence spectra at pH = 1.0 (0.1 M HCl), 7.4 (1× TAE) and 13.0 (0.1 M NaOH) for compounds: (**A**) compound **1b** and **1b_CD**; (**B**) compound **1c** and **1c_CD**, Figure S8: Examples of molecular docking models of compound **1b** in complex with β-CD showing the possibility of the 1:1 (**A**) and 1:2 (**B**) inclusion complexes formation, Figure S9: Examples of molecular docking models of compound **1c** in complex with β-CD showing the possibility of the 1:1 (**A**) and 1:2 (**B**) inclusion complexes formation, Figure S10: Compound **1b_CD** uptake into HeLa cells after 15 min and 24 hours incubation, Figure S11: Compound **1c_CD** uptake into HeLa cells after 15 min and 24 hours incubation, Figure S12: Examples of images for intracellular distribution of compound **1b_CD** after 24 hours (**A**) compared to LysoTracker Red (**B**) and corresponding overlay (**C**), Figure S13: Examples of images for intracellular distribution of compound **1c_CD** after 24 h (**A**) compared to LysoTracker Red (**B**) and corresponding overlay (**C**), Figure S14: Examples of images for intracellular distribution of compound **1b_CD** after 24 hours (**A**) compared to MitoTracker Red (**B**) and corresponding overlay (**C**), Figure S15: Examples of images for intracellular distribution of compound **1c_CD** after 24 hours (**A**) compared to MitoTracker Red (**B**) and corresponding overlay (**C**).
