# Supplementary material for: Cyclodextrin Encapsulated pH Sensitive Dyes as Fluorescent Cellular Probes: Self-Aggregation and In Vitro Assessments
Source: Molecules. 2020 Sep 24;25(19):4397. doi: 10.3390/molecules25194397 (PMC7582577; doi:10.3390/molecules25194397)
Supplement: Supplementary file 1 [file molecules-25-04397-s001.pdf]

Cyclodextrin encapsulated pH sensitive dyes as fluorescent cellular probes: self-aggregation and in vitro assessments

Monica Sardaru [1,2], Oana Carp [1], Elena Laura Ursu [1], Anda-Mihaela Craciun [1], Corneliu Cojocaru [1], Mihaela Silion [1], Vladyslava Kovalska [3,4], Ionel Mangalagiu [2], Ramona Danac [2] and Alexandru Rotaru [1,].*

[1] *"Petru Poni" Institute of Macromolecular Chemistry, Romanian Academy, Grigore Ghica Voda Alley 41 A, 700487 Iasi, Romania*

[2] *Alexandru Ioan Cuza University of Iasi, Chemistry Department, 14 Carol I, 700506 Iasi, Romania*

[3] *Institute of Molecular Biology and Genetics, NASU, 150 Zabolotnogo St., 03143 Kyiv, Ukraine*

[4] *Scientific Services Company Otava Ltd, 150 Zabolotnogo St., 03143 Kyiv, Ukraine*

* Correspondence: rotaru.alexandru@icmpp.ro;

Content

1. ESI-MS spectra – **Figures S1, S2, S3**
2. TEM images – **Figure S4**
3. UV-Vis spectra – **Figures S5, S6**
4. Fluorescence spectra – **Figure S7**
5. Molecular docking models – **Figures S8, S9**
6. Compounds uptake into HeLa cells – **Figures S10, S11**
7. Compounds intracellular distribution and co-staining with LysoTracker Red – **Figures S12, S13**
8. Compounds intracellular distribution and co-staining with MitoTracker Red – **Figures S14, S15**

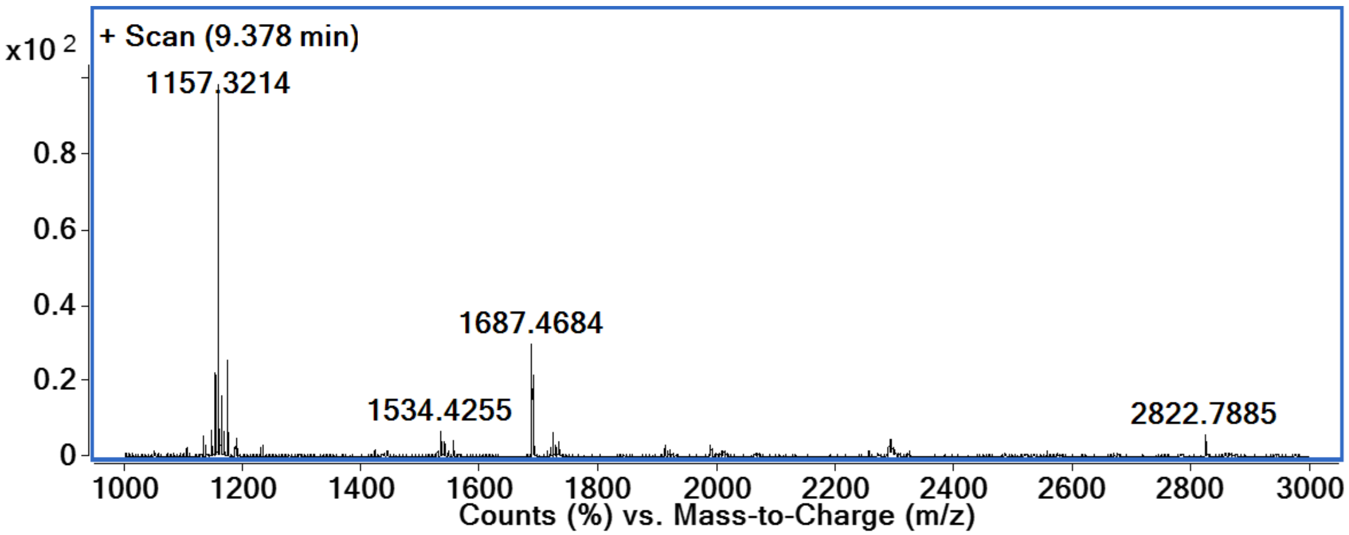
x10 2
+ Scan (9.378 min)
1157.3214
1687.4684
1534.4255
2822.7885
0.8
0.6
0.4
0.2
0
1000
1200
1400
1600
1800
2000
2200
2400
2600
2800
3000
Counts (%) vs. Mass-to-Charge (m/z)

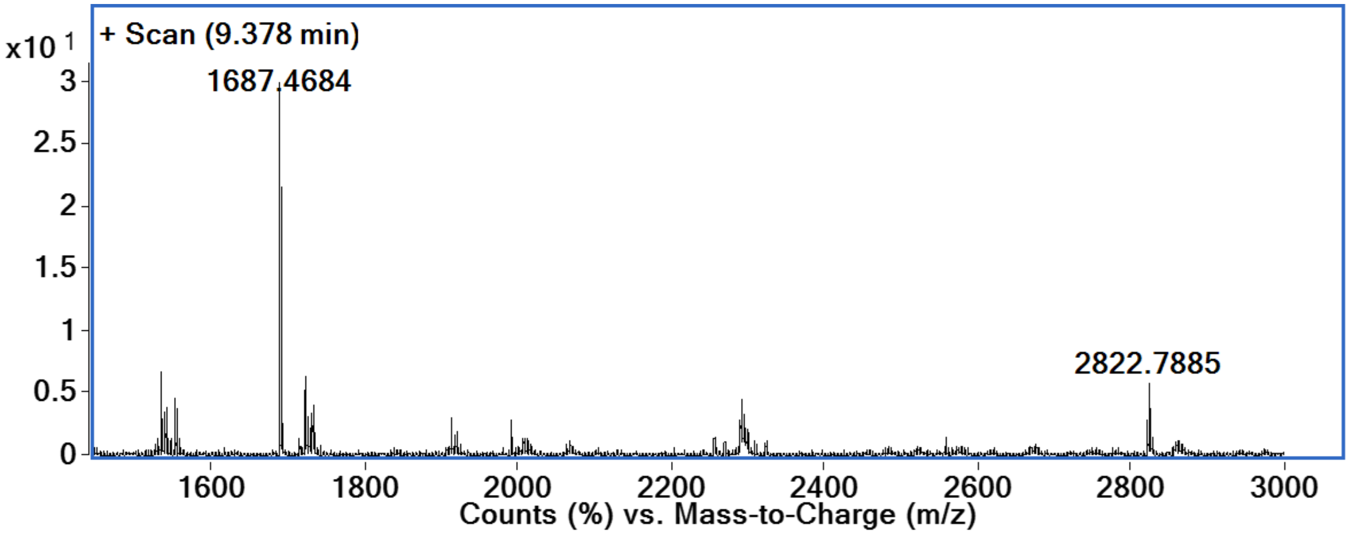
x10 1
+ Scan (9.378 min)
1687.4684
2822.7885
3
2.5
2
1.5
1
0.5
0
1600
1800
2000
2200
2400
2600
2800
3000
Counts (%) vs. Mass-to-Charge (m/z)

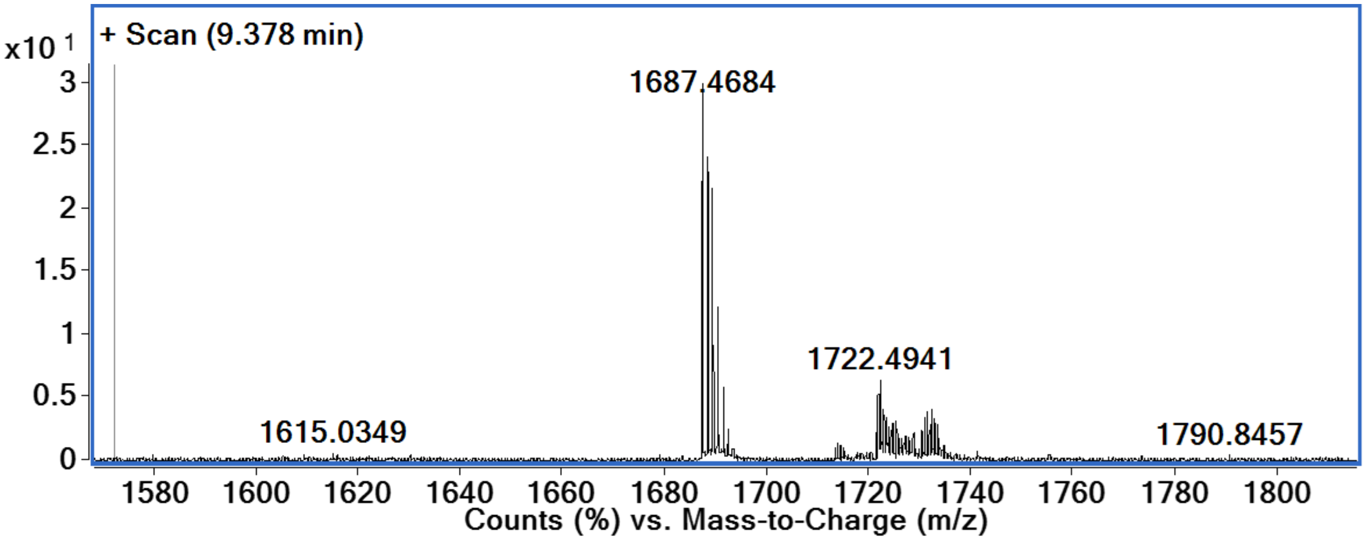
x10 1
+ Scan (9.378 min)
1687.4684
1722.4941
1615.0349
1790.8457
3
2.5
2
1.5
1
0.5
0
1580
1600
1620
1640
1660
1680
1700
1720
1740
1760
1780
1800
Counts (%) vs. Mass-to-Charge (m/z)

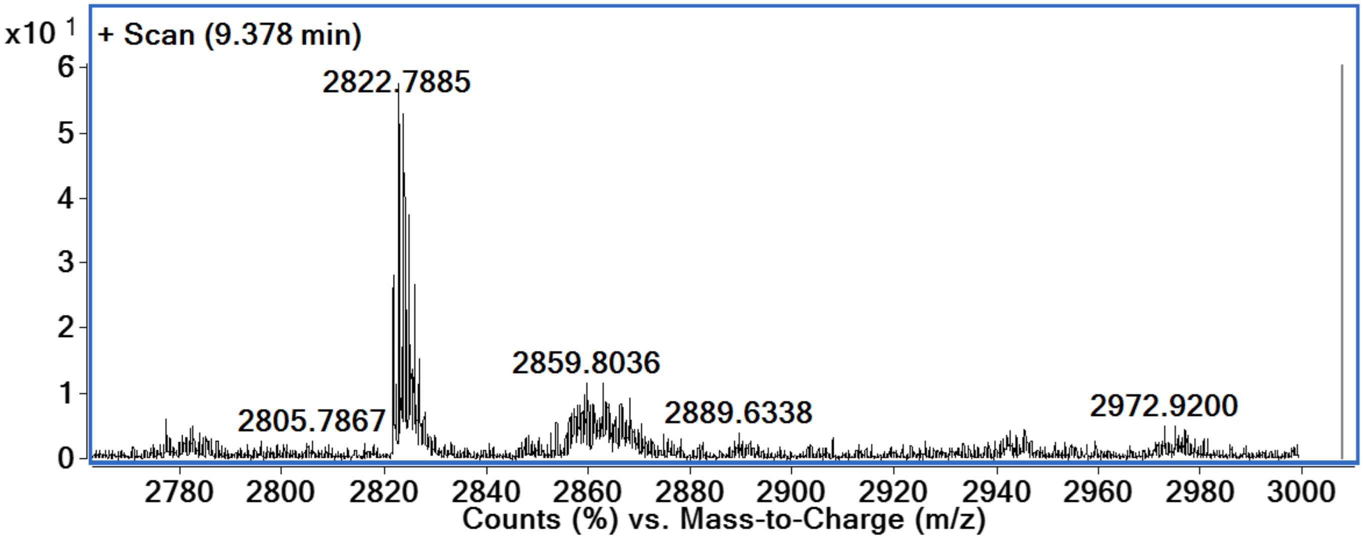

Figure S1. Examples of ESI-MS spectra of the **1a_CD** at 1:5 molar ratio between indolizine **1a** and CD. Peaks corresponding to: β-CD (MNa^+ ion at *m/z* 1157), the formation of 1:1 inclusion complex (M^+-Br + CD ion at *m/z* 1687) and 1:2 (M^+-Br + 2CD ion at *m/z* 2822) have been identified.

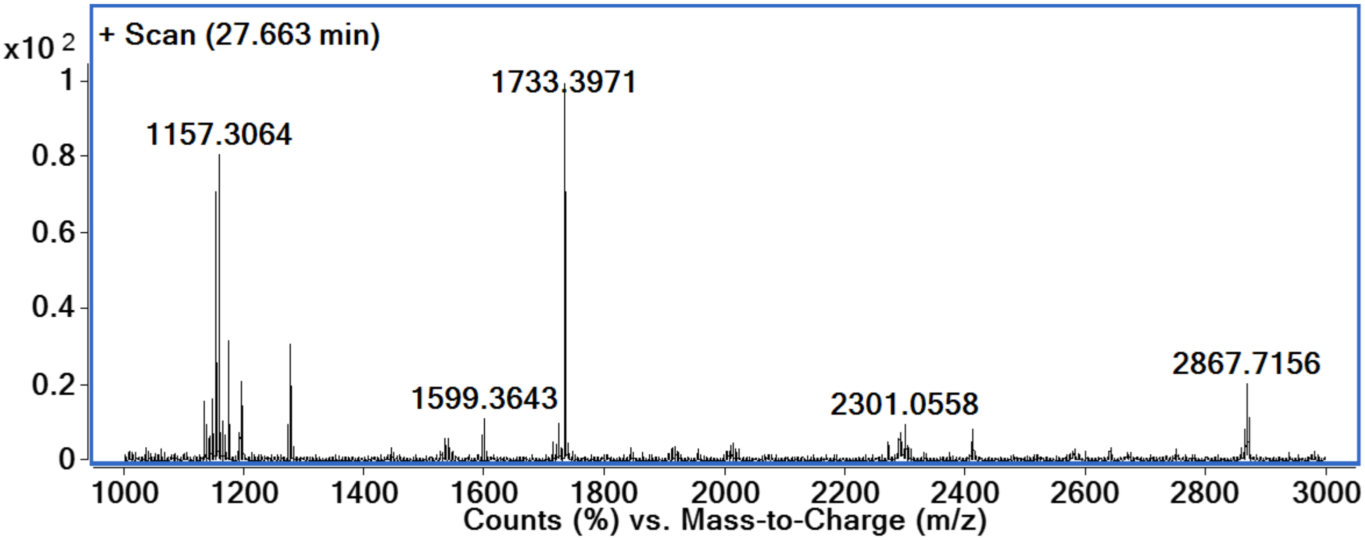
+ Scan (27.663 min)
x10 2
1
0.8
0.6
0.4
0.2
0
1157.3064
1733.3971
1599.3643
2301.0558
2867.7156
1000
1200
1400
1600
1800
2000
2200
2400
2600
2800
3000
Counts (%) vs. Mass-to-Charge (m/z)

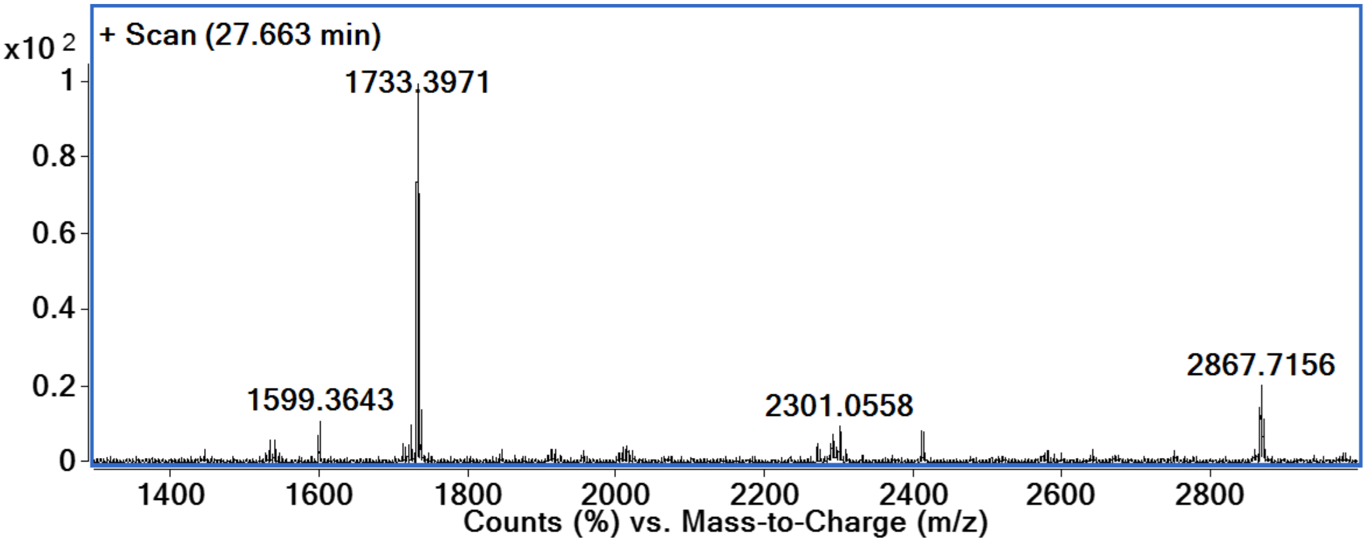
+ Scan (27.663 min)
x10 2
1
0.8
0.6
0.4
0.2
0
1733.3971
1599.3643
2301.0558
2867.7156
1400
1600
1800
2000
2200
2400
2600
2800
Counts (%) vs. Mass-to-Charge (m/z)

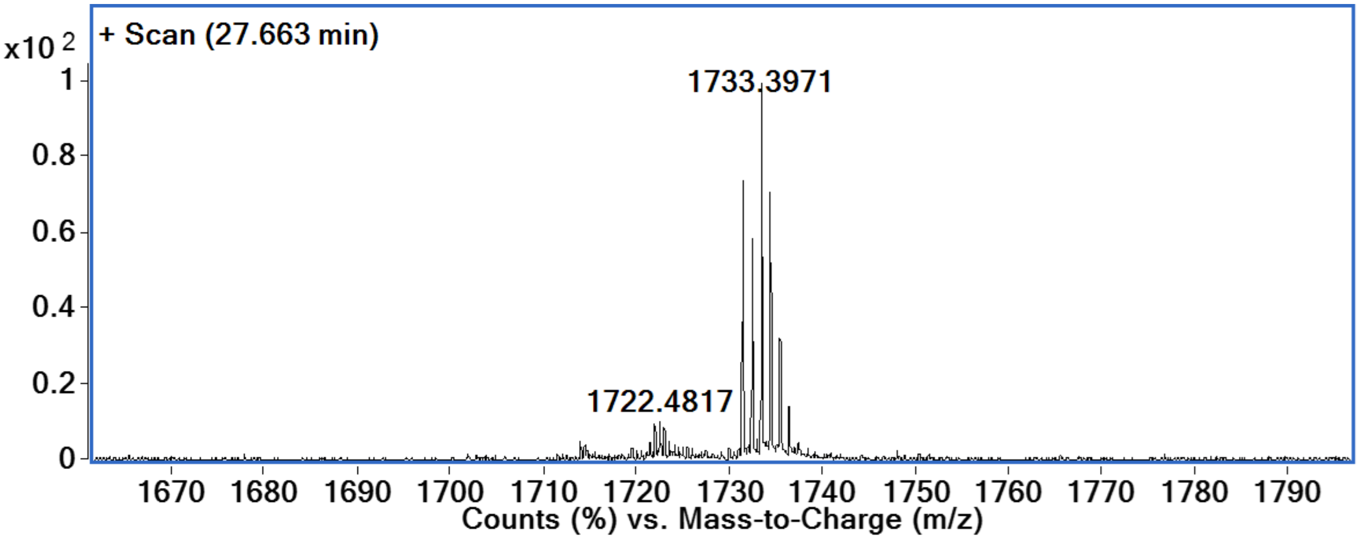
+ Scan (27.663 min)
x10 2
1
0.8
0.6
0.4
0.2
0
1733.3971
1722.4817
1670
1680
1690
1700
1710
1720
1730
1740
1750
1760
1770
1780
1790
Counts (%) vs. Mass-to-Charge (m/z)

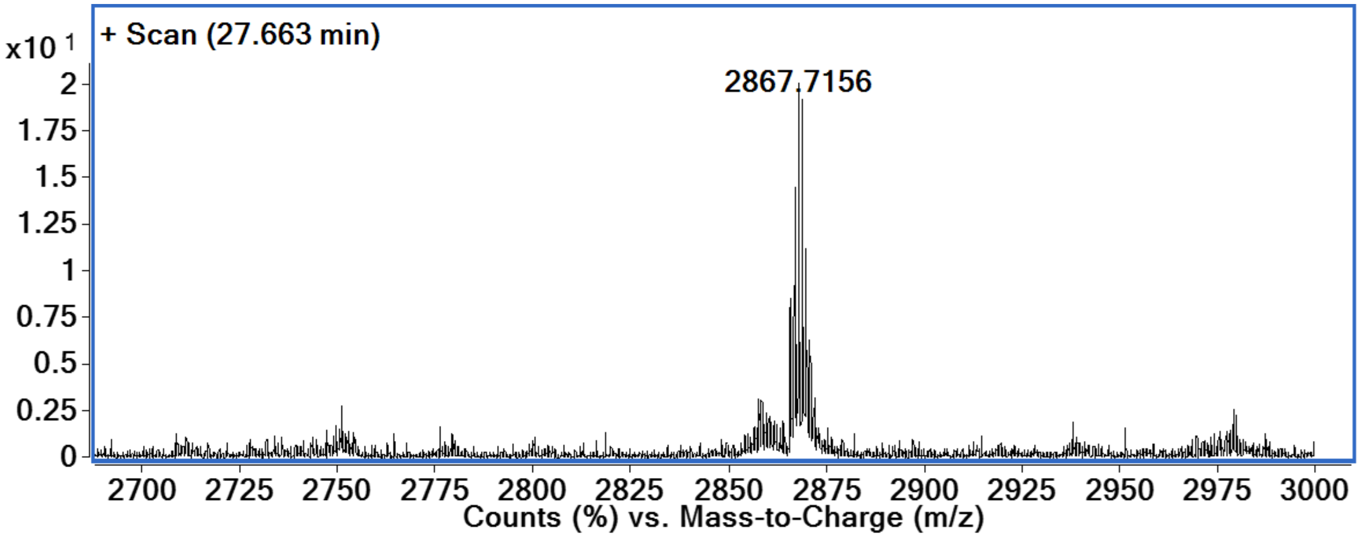

Figure S2. Examples of ESI-MS spectra of the **1b_CD** at 1:5 molar ratio between indolizine **1b** and CD. Peaks corresponding to: β-CD (MNa^+ ion at *m/z* 1157), the formation of 1:1 inclusion complex (M^+-Br + CD ion at *m/z* 1733) and 1:2 (M^+-Br + 2CD ion at *m/z* 2867) have been identified.

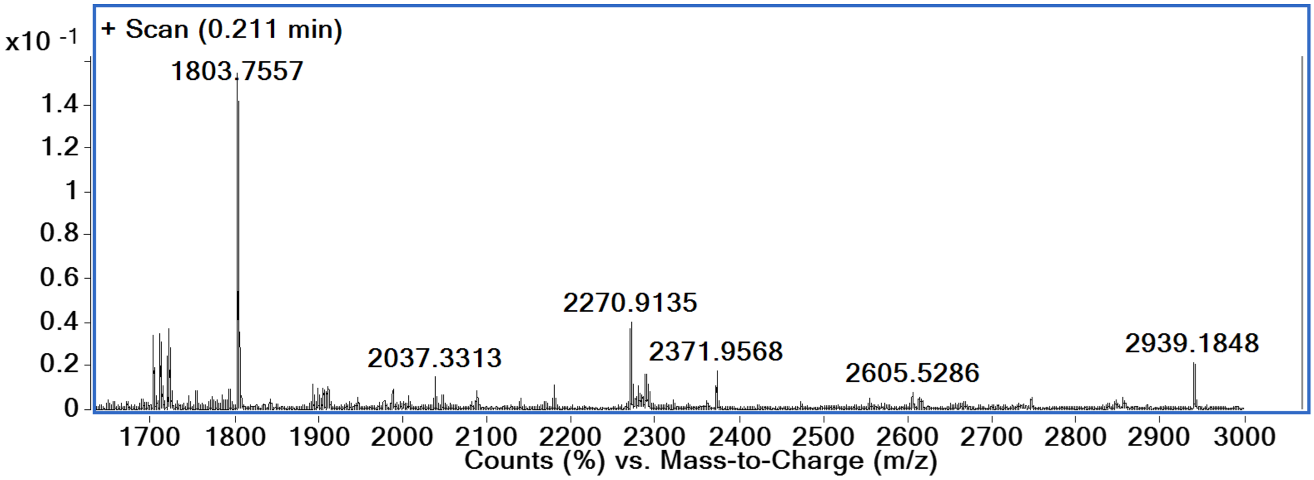

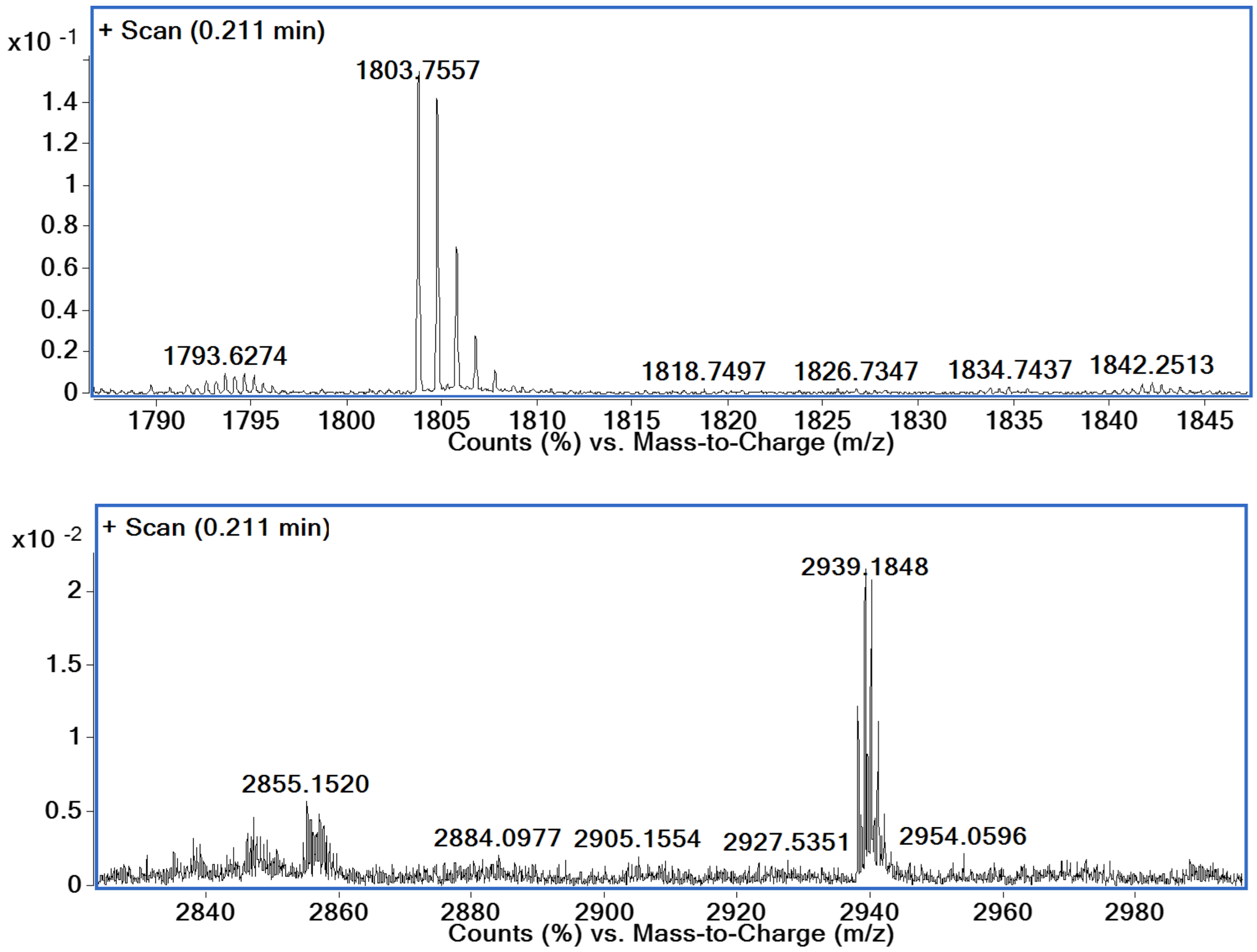

Figure S3. Examples of ESI-MS spectra of the **1c_CD** at 1:5 molar ratio between indolizine **1c** and CD. Peaks corresponding to: the formation of 1:1 inclusion complex (M^+-Br + CD ion at *m/z* 1803) and 1:2 (M^+-Br + 2CD ion at *m/z* 2939) have been identified.

(A)

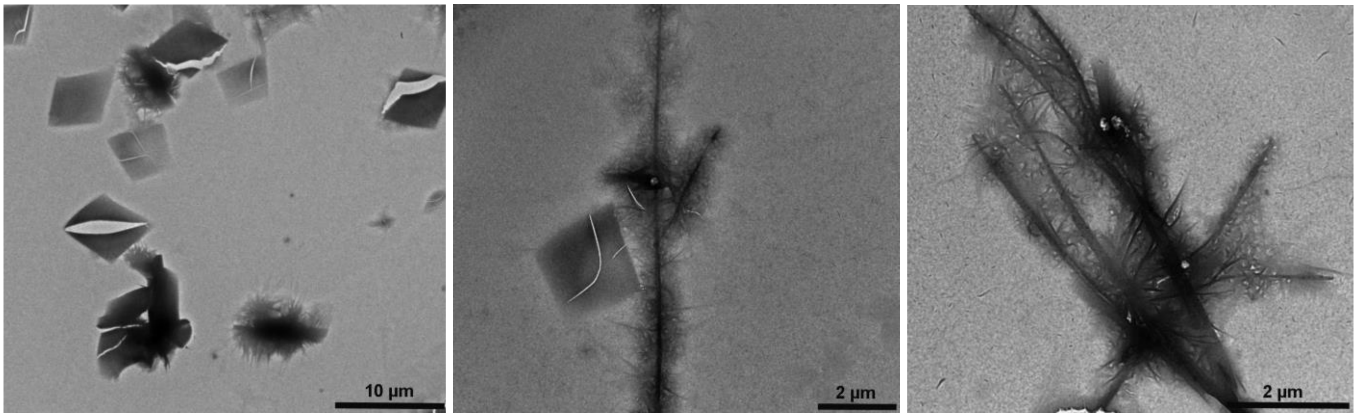

(B)

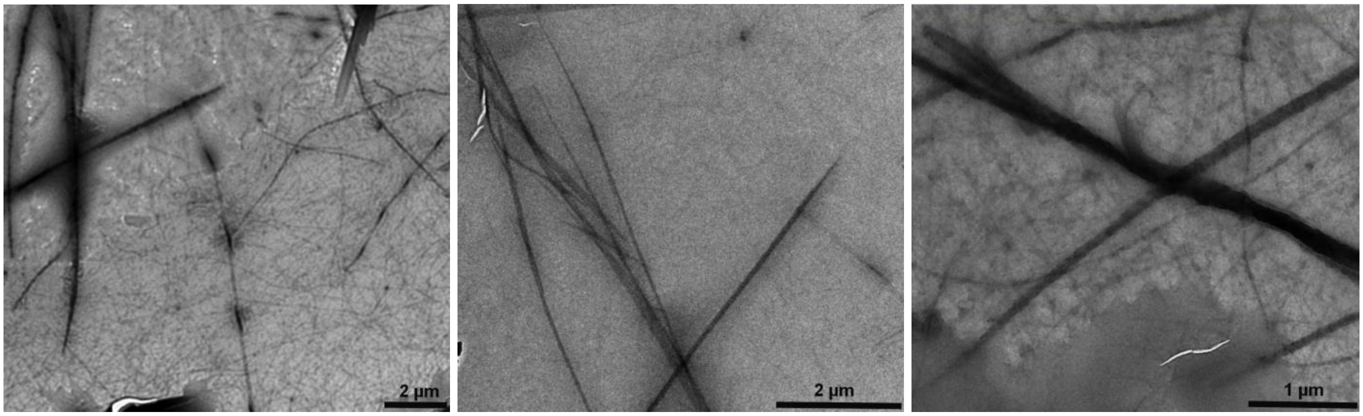

(C)

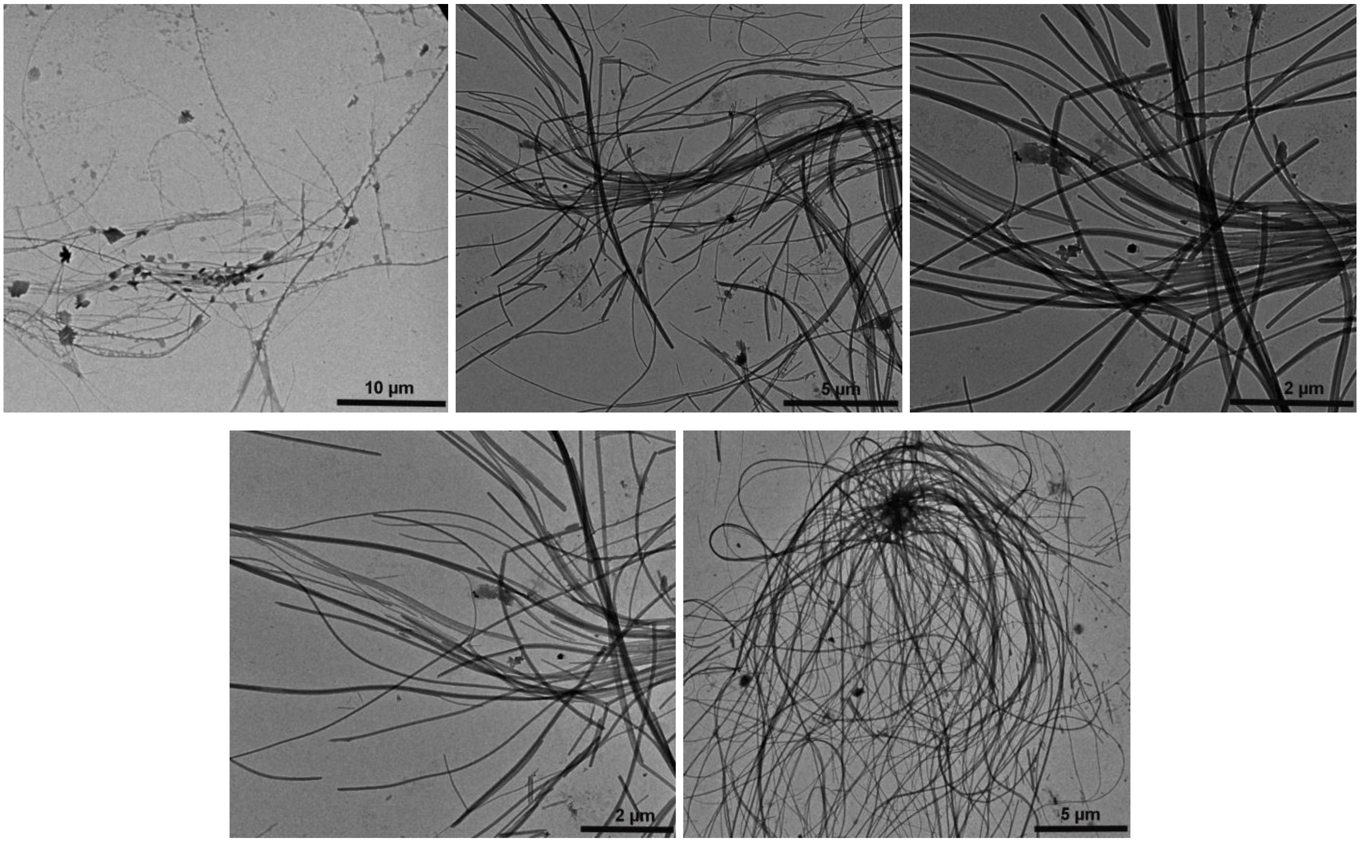

Figure S4: Examples of TEM images for compounds **1a_CD** (**A**); **1b_CD** (**B**); **1c_CD** (**C**).

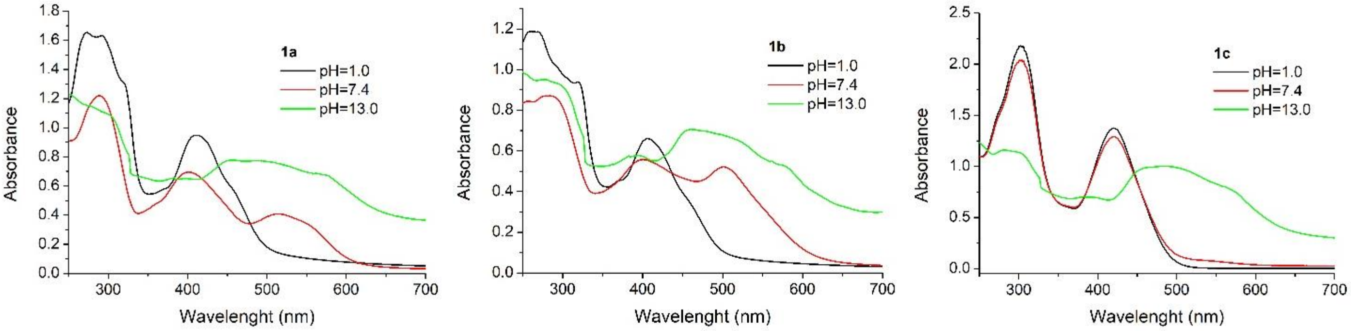

Figure S5: UV-Vis spectra of compounds **1(a-c)** at pH value of 1.0 (0.1 M HCl), 7.4 (1xTAE) and 13.0 (0.1 M NaOH).

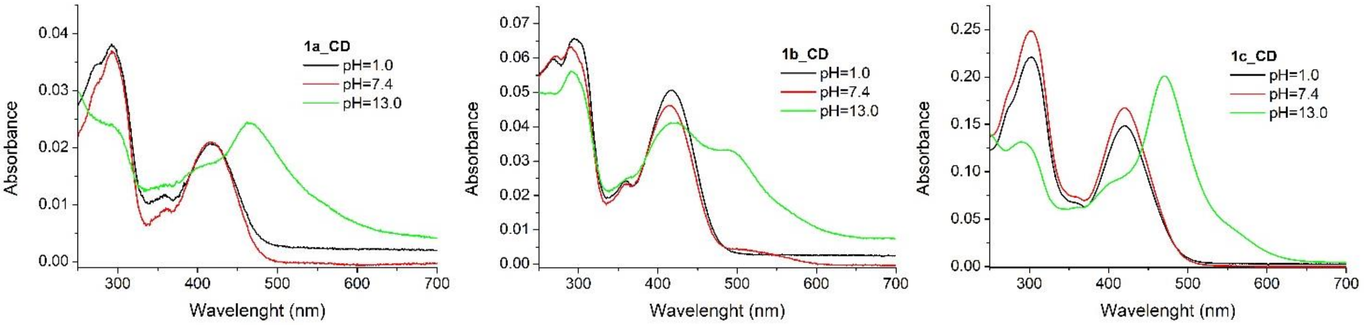

Figure S6: UV-Vis spectra of compounds **1(a-c)_CD** at pH value of 1.0 (0.1 M HCl), 7.4 (1xTAE) and 13.0 (0.1 M NaOH).

(A)

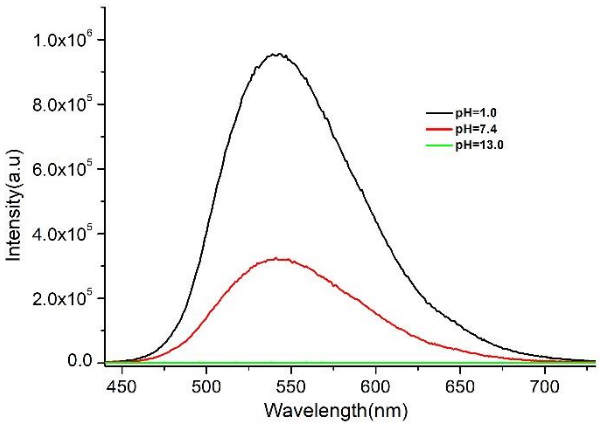

(B)

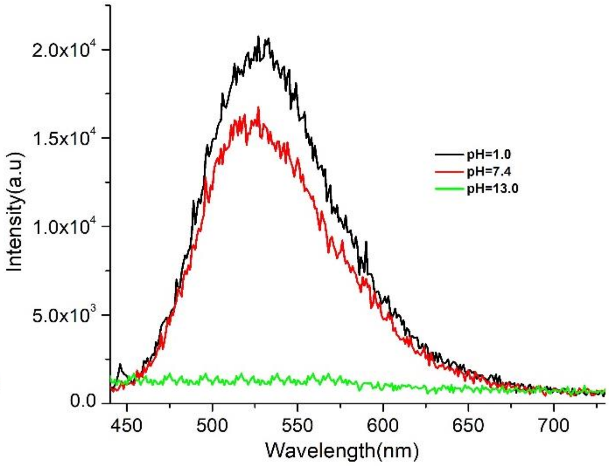

(C) (D)

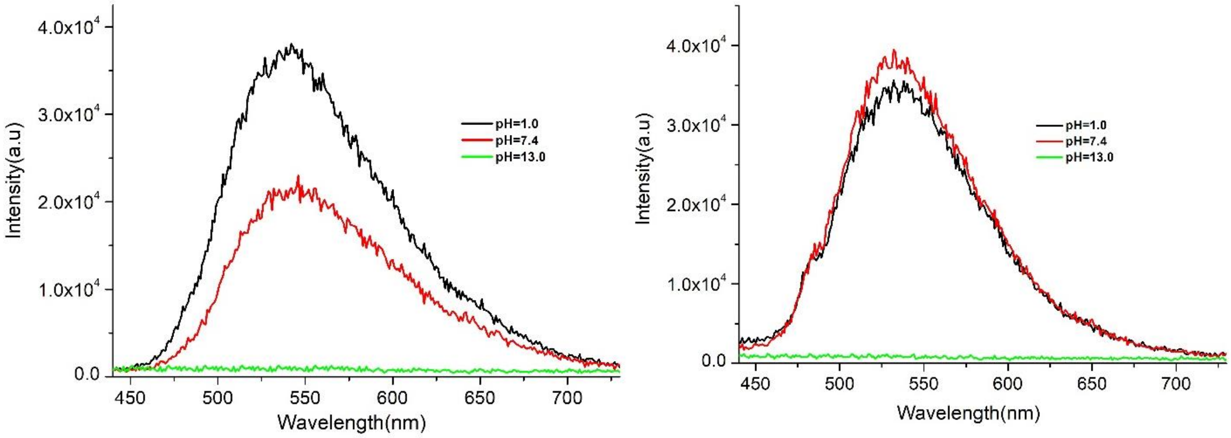

Figure S7: Fluorescence spectra at pH = 1.0 (0.1 M HCl), 7.4 (1xTAE) and 13.0 (0.1 M NaOH)for compounds: **(A)** compound **1b**, **(B)**compound **1b_CD**, **(C)** compound **1c** and **(D)** compound **1c_CD**.

(**A**) **(B)**

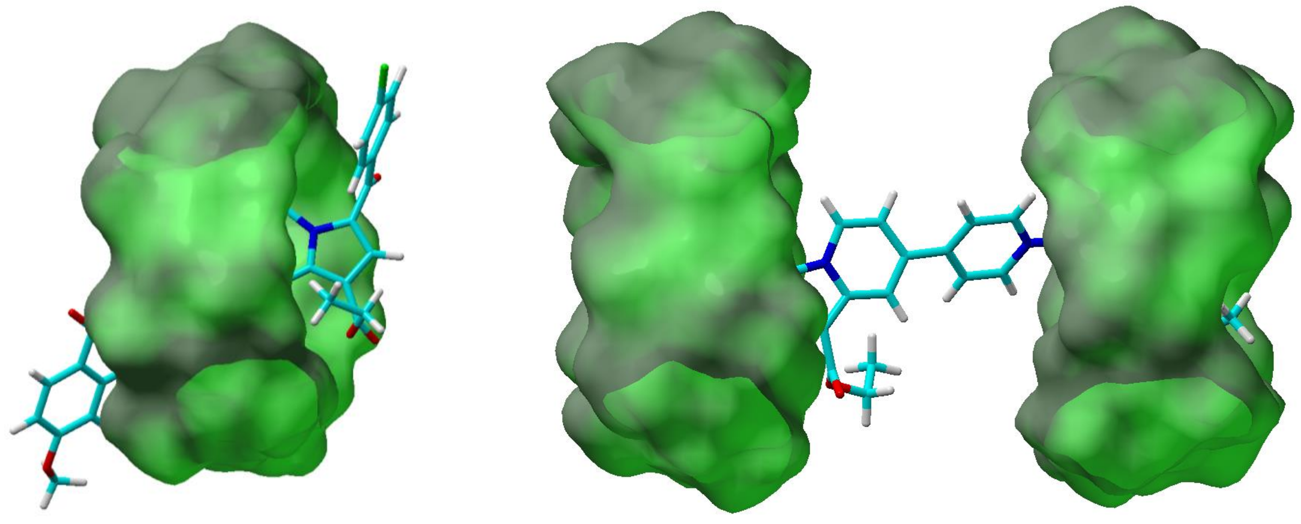

Figure S8: Examples of molecular docking models of compound **1b** in complex with β-CD showing the possibility of the 1:1 (**A**) and 1:2 (**B**) inclusion complexes formation**.**

(**A**) **(B)**

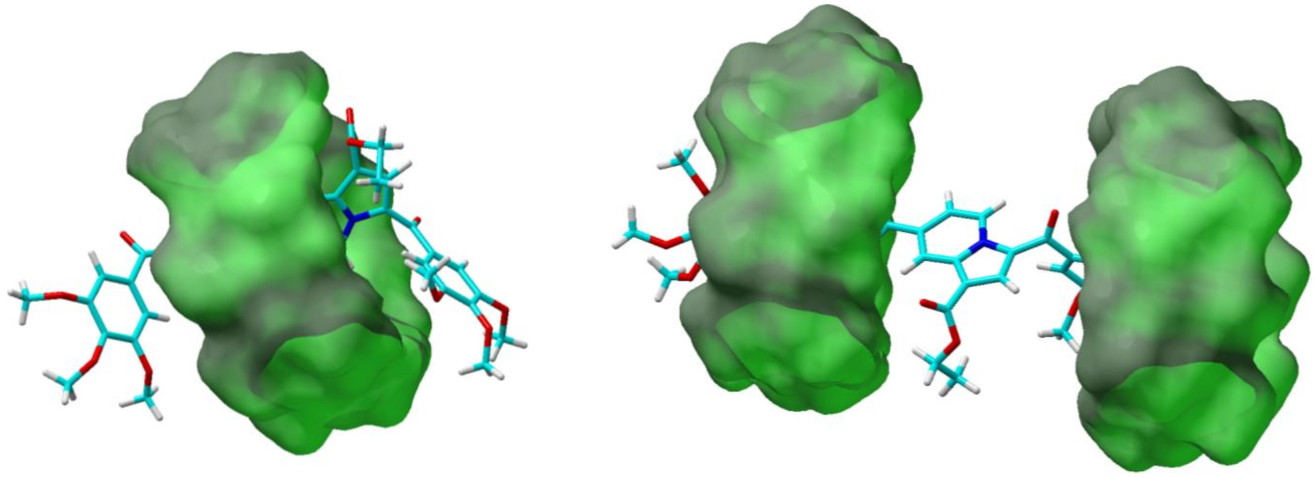

Figure S9: Examples of molecular docking models of compound **1c** in complex with β-CD showing the possibility of the 1:1 (**A**) and 1:2 (**B**) inclusion complexes formation**.**

A B

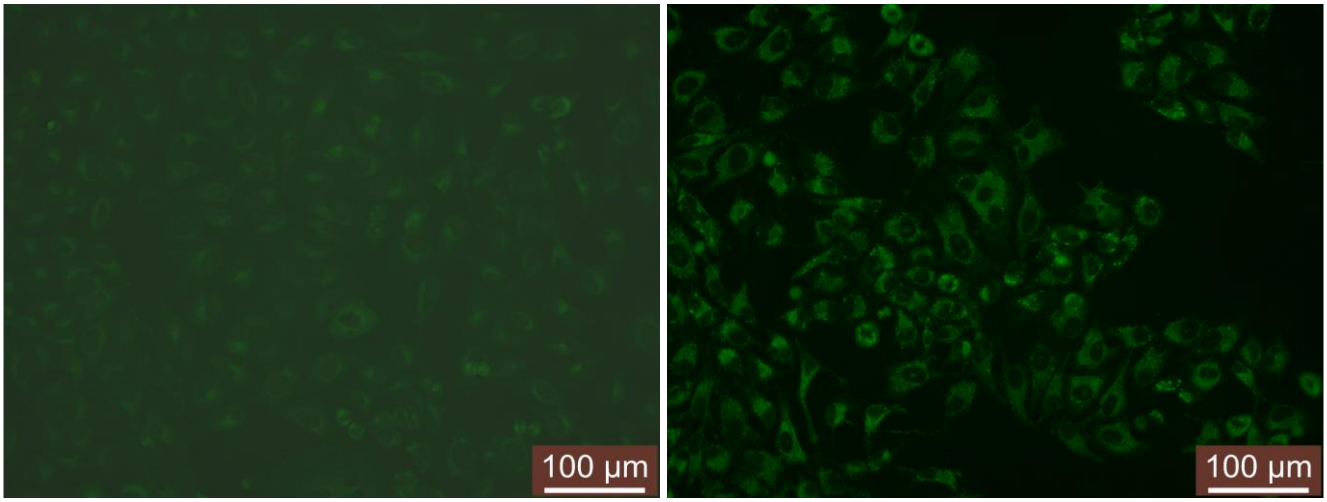

Figure S10. Compound **1b_CD** uptake into HeLa cells after 15 min (left) and 24 hours (right) incubation.

A B

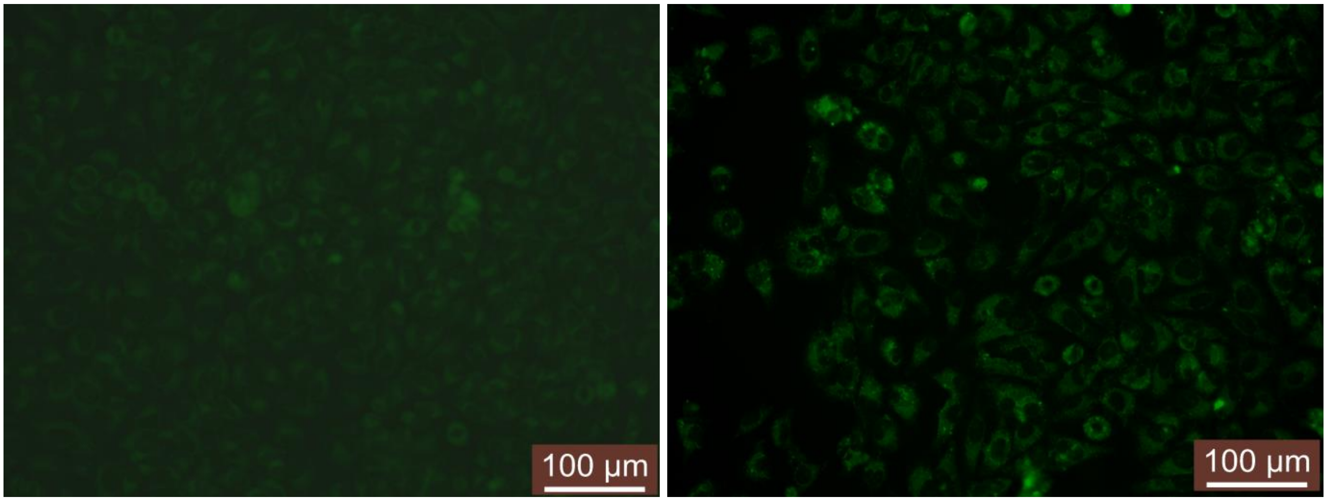

Figure S11. Compound **1c_CD** uptake into HeLa cells after 15 min (left) and 24 hours (right) incubation.

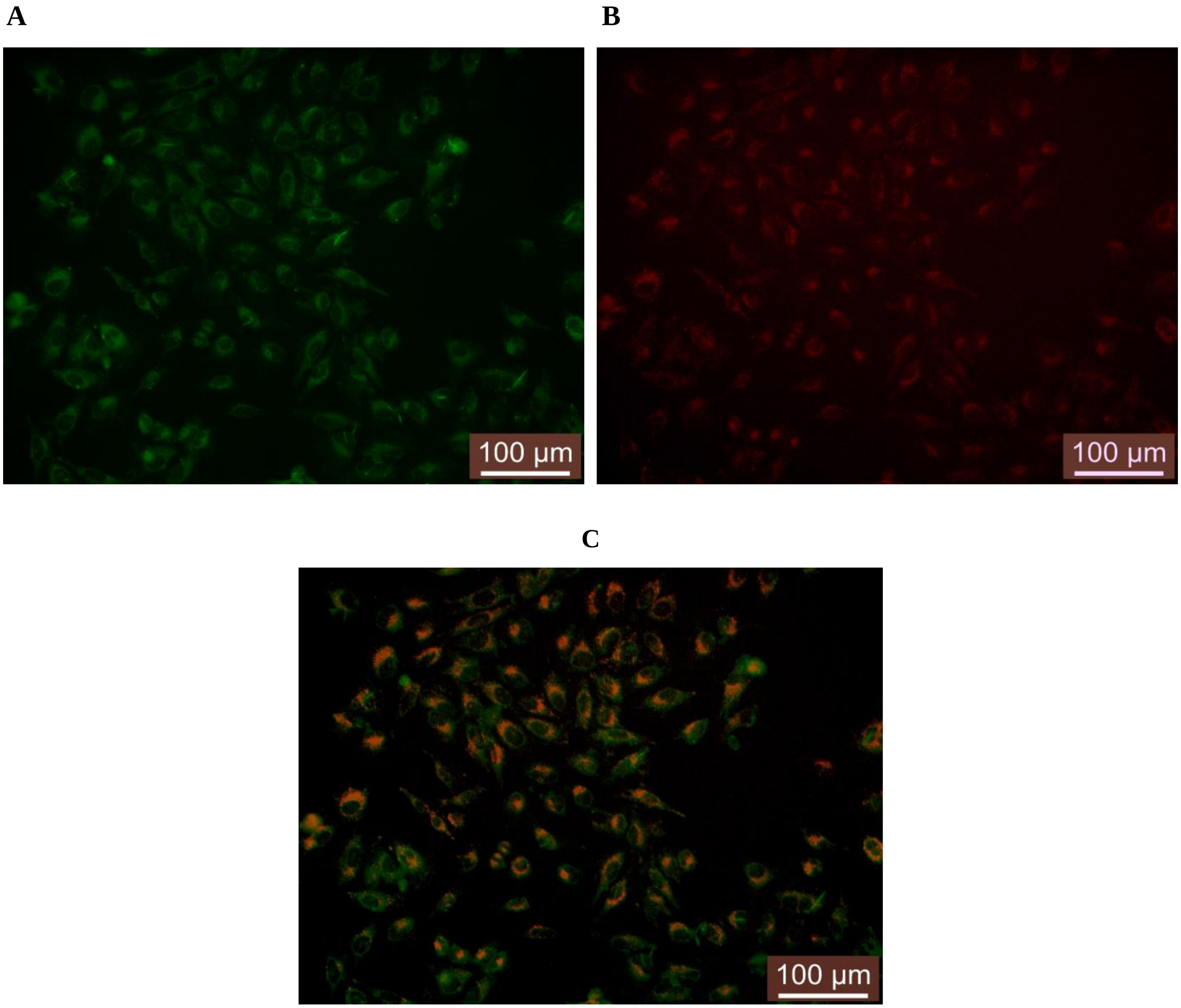

Figure S12. Examples of images for intracellular distribution of compound **1b_CD** after 24 hours (A) compared to LysoTracker Red (B) and corresponding overlay (C).

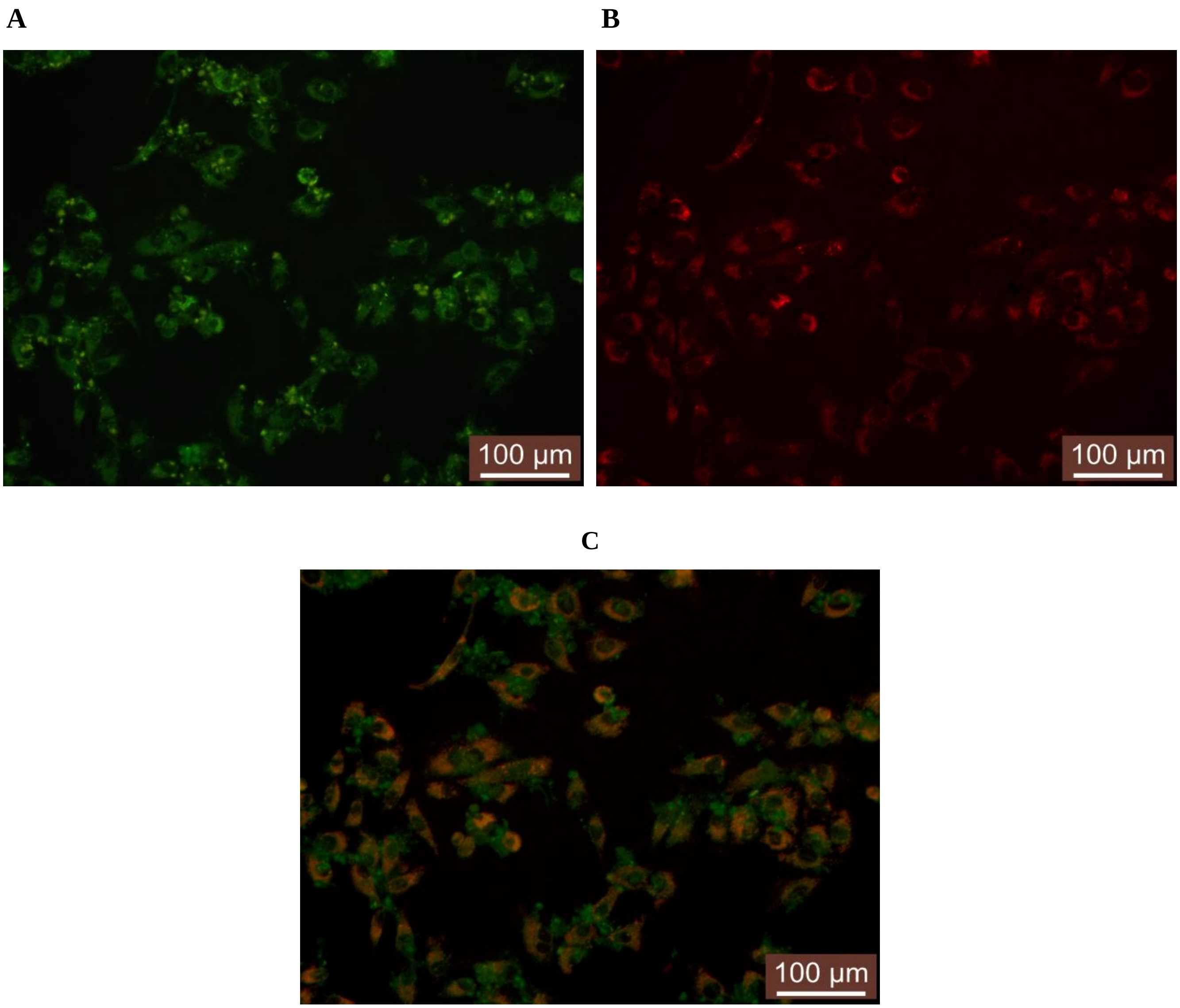

Figure S13. Examples of images for intracellular distribution of compound **1c_CD** after 24 hours (A) compared to LysoTracker Red (B) and corresponding overlay (C).

A B C

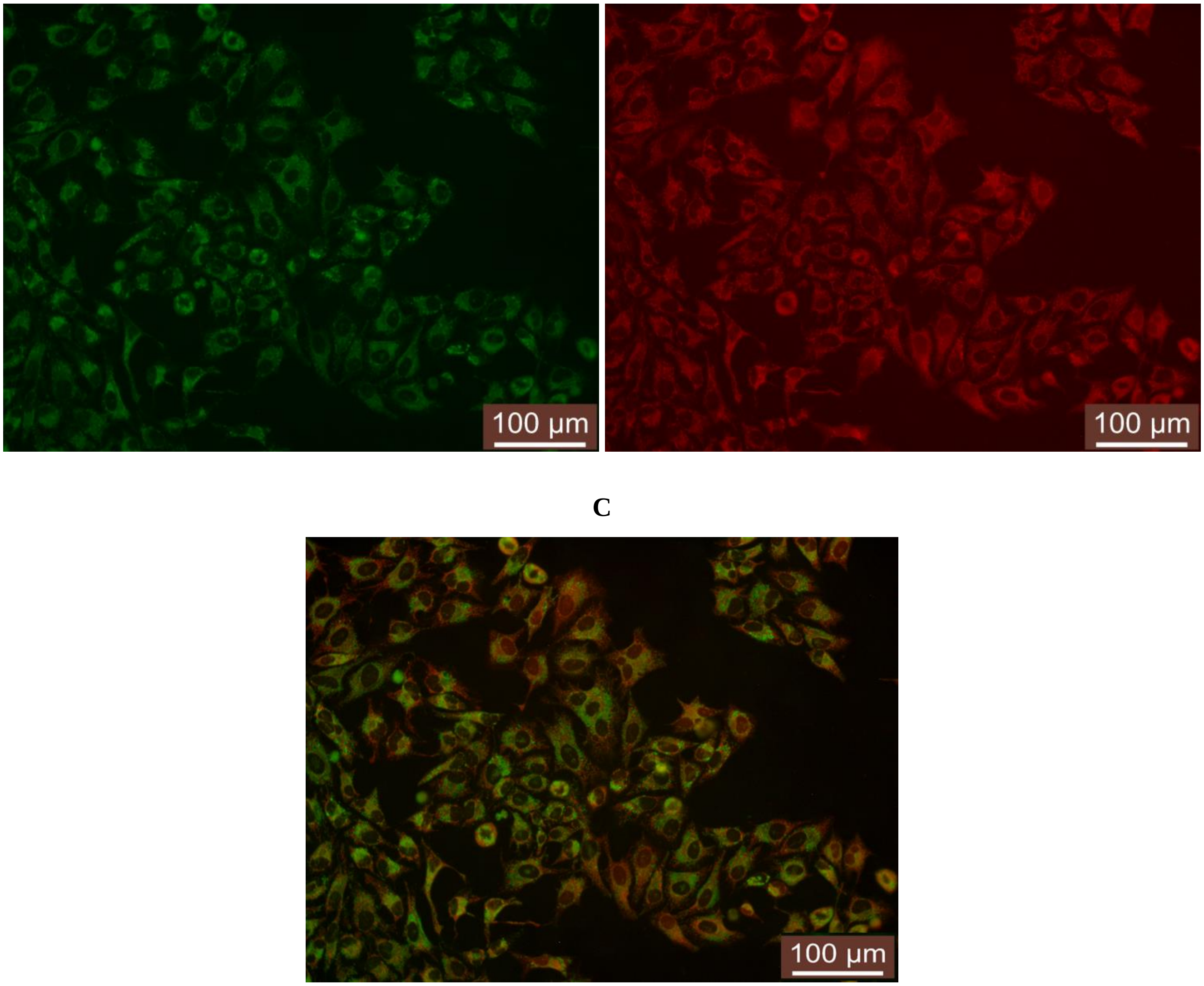

Figure S14. Examples of images for intracellular distribution of compound **1b_CD** after 24 hours (A) compared to MitoTracker Red (B) and corresponding overlay (C).

A B

C

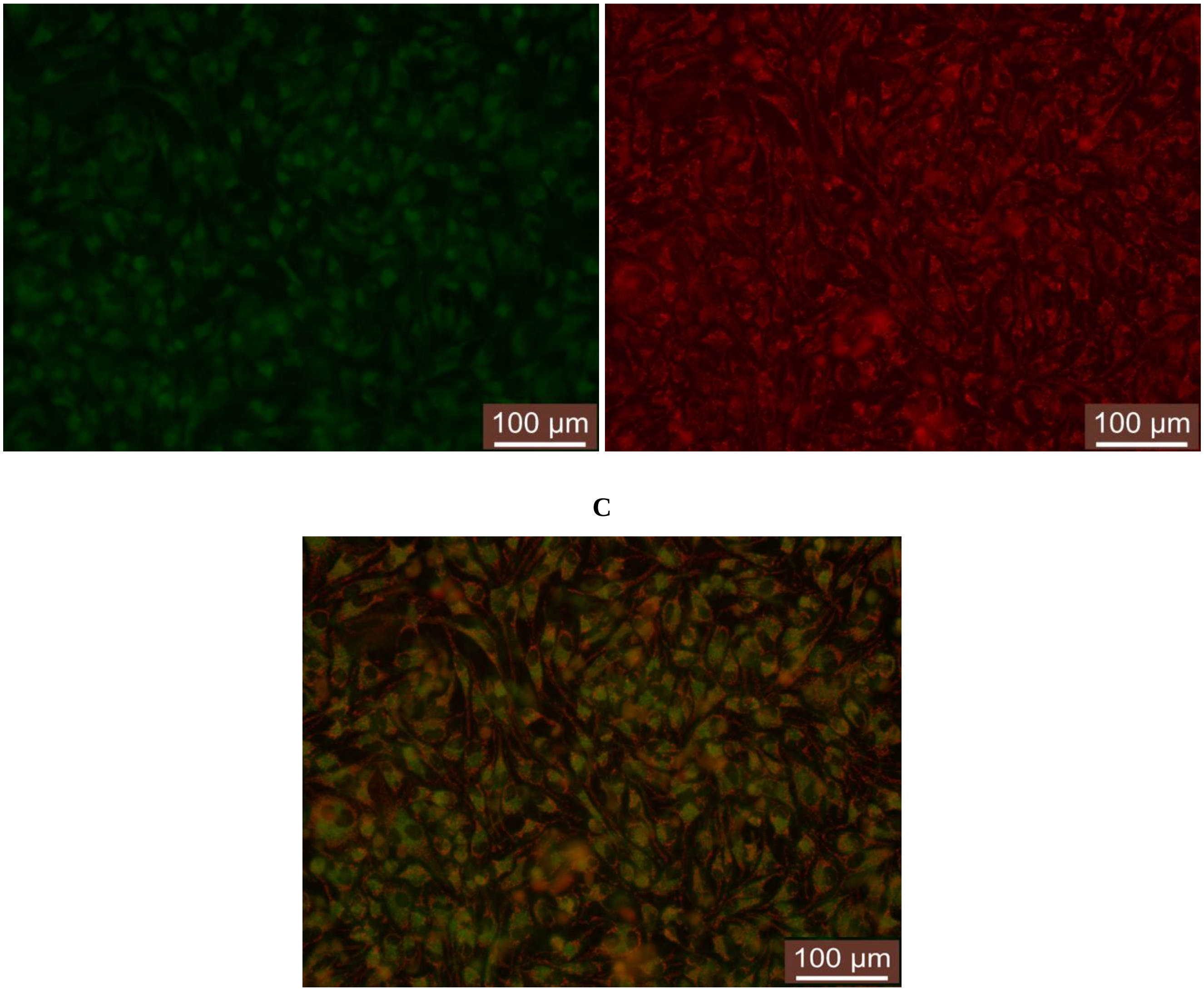

Figure S15. Examples of images for intracellular distribution of compound **1c_CD** after 24 hours (A) compared to MitoTracker Red (B) and corresponding overlay (C).
